# Chemostat culture systems support diverse bacteriophage communities from human feces

**DOI:** 10.1186/s40168-015-0124-3

**Published:** 2015-11-09

**Authors:** Tasha M. Santiago-Rodriguez, Melissa Ly, Michelle C. Daigneault, Ian H. L. Brown, Julie A. K. McDonald, Natasha Bonilla, Emma Allen Vercoe, David T. Pride

**Affiliations:** Department of Pathology, University of California, 9500 Gilman Drive, MC 0612, La Jolla, CA 92093-0612 USA; Department of Molecular and Cellular Biology, University of Guelph, Guelph, Ontario Canada; Department of Biology, San Diego State University, San Diego, CA USA; Department of Medicine, University of California, San Diego, CA USA

**Keywords:** Stool, Feces, Bacteriophage, Microbiome, Virome, Metagenome, Chemostat, Viral communities

## Abstract

**Background:**

Most human microbiota studies focus on bacteria inhabiting body surfaces, but these surfaces also are home to large populations of viruses. Many are bacteriophages, and their role in driving bacterial diversity is difficult to decipher without the use of in vitro ecosystems that can reproduce human microbial communities.

**Results:**

We used chemostat culture systems known to harbor diverse fecal bacteria to decipher whether these cultures also are home to phage communities. We found that there are vast viral communities inhabiting these ecosystems, with estimated concentrations similar to those found in human feces. The viral communities are composed entirely of bacteriophages and likely contain both temperate and lytic phages based on their similarities to other known phages. We examined the cultured phage communities at five separate time points over 24 days and found that they were highly individual-specific, suggesting that much of the subject-specificity found in human viromes also is captured by this culture-based system. A high proportion of the community membership is conserved over time, but the cultured communities maintain more similarity with other intra-subject cultures than they do to human feces. In four of the five subjects, estimated viral diversity between fecal and cultured communities was highly similar.

**Conclusions:**

Because the diversity of phages in these cultured fecal communities have similarities to those found in humans, we believe these communities can serve as valuable ecosystems to help uncover the role of phages in human microbial communities.

**Electronic supplementary material:**

The online version of this article (doi:10.1186/s40168-015-0124-3) contains supplementary material, which is available to authorized users.

## Background

Human body surfaces are inhabited by diverse viral communities, and the majority of those identifiable viruses are bacteriophages [1, 2]. The most well studied viral communities to date are those in the human gut and the oral cavity. Viruses in the gut are rapidly evolving [3], exhibiting subject-specificity [4], responding to dietary changes, and persisting over time [5]. Similarly, viruses in the oral cavity are highly personalized [6] and highly persistent, possibly as a result of their ability to evade defense mechanisms utilized by oral bacteria [6–8]. The oral cavity has different biogeographic sites with specific viral communities whose membership differs significantly in periodontal health and disease [9]. Limitations due to virus assembly from metagenome data, however, often result in overestimations of the diversity present in human viral communities [10]. There also are viral communities inhabiting human skin [11] and the respiratory tract [12], but the role of viruses as members of the human microbial communities is not well understood.

Bacteriophages possess the capacity to alter microbial communities by either lysing their hosts or providing phenotypic advantages to recipient bacteria [13–15]. Phages may influence biogeochemical cycles in aquatic environments by decreasing the relative abundances of specific bacterial species [16, 17] or by supporting bacterial populations with the wide repertoire of metabolic-associated genes they possess [18, 19]. The ability of phages to influence microbial diversity has been hypothesized to have consequences for human health by altering the normal microbiota that may have protective effects against colonization by more pathogenic microorganisms. While some phages have lysogenic lifestyles and may contribute gene functions to their hosts in human microbial communities, others have lytic lifestyles and may be responsible for driving microbial diversity on human body surfaces. The subgingival crevice in periodontal disease is enriched for myoviruses [9] that typically have lytic lifestyles, which implicates them as potential drivers of microbial diversity in human disease. That microbial diversity in human disease that may be potentiated by viruses provides a significant rationale for more studies focused on the role of viruses as members of human microbial communities.

Studies of microbiota can be limited due to restrictions imposed by working with human subjects [20]. For example, examining the effects of a lytic virus on human microbiota meets with ethical challenges that limit experimental design. Some studies have been valuable in discerning the effects of perturbations on human gut microbiota, but personalized individual microbial profiles and individual confounders such as age, diet, and medications may influence gut microbial composition [21–26]. The restrictions associated with working with human subjects and the potential for significant confounders despite well-designed experiments necessitate the development of cultivation-based systems capable of reproducing the complex interactions of the microbiota on human body surfaces. There currently are no known cultivation-based ecosystems that effectively reproduce the diversity of viral populations in humans.

Model microbial systems must be reproducible, highly stable, and must retain similar levels of diversity to the inocula from which they were derived [27, 28]. Interactions between host and phage have been previously modeled in gnotobiotic mice [29], which allowed for the tracking of phage dynamics with their individual hosts. The cellular microbiota of the human gut has been modeled by cultivating fecal samples in complex chemostat systems [20, 30]. These microbial communities reach an equilibrium that resembles the gut community structure from which they originated [27, 31–33]. Models of the oral microbiota in other culture-based systems also have been shown to reproduce much of the taxonomical and functional characteristics of the oral biofilm [34]. These systems offer the potential to study the response of microorganisms to perturbations in controllable and reproducible environments that reduce the potential for confounders that often are encountered working with human subjects [20, 35]. None of these cultivation-based ecosystems have been shown to be inhabited by viral communities, but researchers previously have successfully cultivated individual viruses in these types of systems [36, 37]. We believe that because chemostat culture systems effectively reproduce much of the cellular microbiota in the gut, they may also be home to substantial viral communities. The goals of this study were as followings: (1) to demonstrate that cultured fecal microbial communities are home to robust viral communities, (2) to develop techniques to investigate the diversity of the viral communities in cultured fecal communities, and (3) to determine whether cultured fecal communities have similarities to viral communities present in human feces.

## Results

### Human subjects and chemostat cultured communities

We recruited five human subjects through the University of Guelph and sampled their feces. Donors #1, #2, and #10 were a co-habiting family unit of father, mother, and child, respectively, while donors #8 and #9 were unrelated. Each fecal sample was homogenized and processed immediately into a chemostat vessel, and the remainder of the feces was stored until processing of the viromes could take place. Chemostat vessels were operated under conditions designed to mimic the human distal colon [30]. Cultured microbial communities were taken from donors #1, #2, and #10 on days 4, 8, 12, 16, and 24 and from donors #8 and #9 on days 3, 6, 12, 18, and 24. Previous studies have shown that cellular microbial communities reach stable state in culture by day 24 [30]. We tested for the presence of fluorescent-staining particles (FSPs) in feces and cultures, similar to those previously described, to indicate the likely presence of viruses in both sample types [38]. On day 24, we found that there were numerous FSPs in both sample types, with a mean 3.7 × 10^9^ FSPs in feces and 1.4 × 10^9^ FSPs in the chemostat cultures for all subjects studied. The presence of such high densities of FSPs in both sample types strongly suggested the presence of substantial viral communities.

We isolated and processed viruses from both feces and chemostat cultures utilizing sequential filtration followed by cesium chloride density gradient centrifugation according to our previously described protocols [39]. We sequenced the resulting viral DNA from the feces and chemostat cultures of the five donors using semiconductor sequencing [40] and produced 18,584,604 reads (619,487 reads per time point and sample type) of mean length 215 nucleotides (Additional file 1: Table S1). We used BLASTN to compare all viromes to the RDP 16S rRNA genes database (E-value <10^−5^) and found that all were free of 16S rRNA gene homologues. We also used BLASTN to search the viromes for homologues against a human reference genome, and some similar sequences (E-value <10^−5^) were identified and removed prior to further analysis. These data suggest that these chemostat and fecal viromes were relatively free of contaminating cellular nucleic acids.

### Identification of viruses and viral families in cultured fecal material

We assembled virome reads from each subject to construct longer contigs, as this generally results in more productive searches for sequence similarities. We used several different assemblers including CLC Genomics Workbench, MetaVelvet [41], and IDBA-UD [42] in the construction of contigs and found that CLC Genomics Workbench produced fewer contigs with longer mean and maximum lengths and higher N50 values than the other assemblers tested (Additional file 2: Table S2). With the CLC assembler, 96.4 ± 1.3 % of all reads were assembled into contigs. Therefore, we utilized the contigs constructed using CLC Genomics Workbench throughout the study. The mean GC content for all contigs was 45.5 % (mean of 46.3 % for cultured viromes and 41.7 % for fecal viromes; *p* = 0.01) (Additional file 3: Figure S1, Panel A). The mean length amongst all contigs was 941 nucleotides (mean of 908 nucleotides for cultured viromes and 965 nucleotides for fecal viromes; *p* = 0.35) (Additional file 3: Figure S1, Panel B). The differences identified in GC content suggest that there may be features of viromes that are specific to both fecal and cultured communities.

**Fig. 1:**
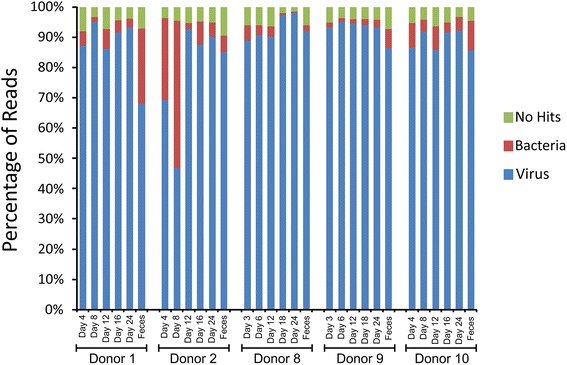
Plots of percentage of virome reads that belong to contigs with significant sequence similarities in the NCBI NR database. The percentage of reads is demonstrated on the Y-axis, and each donor, time point, and sample type is demonstrated on the X-axis

Prior studies of viromes generally have identified large proportions of putative viruses that have no significant sequence similarities in available databases. We used BLASTX analysis of the assembled contigs against the NCBI non-redundant database (NR) to determine the relative proportion of our viromes that had significant sequence similarities. We utilized the numbers of reads assigned to each contig to identify the proportions of reads belonging to contigs with significant sequence similarities in the NR database. We found that in all subjects combined, 94.9 ± 1.7 % of the reads belonged to contigs that had similar sequences in the NR database (Fig. 1); 88.0 ± 10.2 % of those reads belonged to contigs that had sequence similarities to phages, 6.9 ± 9.9 % belonged to contigs that were similar to bacteria, and 5.1 ± 1.7 % belonged to contigs with no significant sequence similarities in the NR database. Most virome studies have contigs that have significant sequence similarities to bacteria; however, in the absence of finding 16S rRNA genes in any of the viromes, the similarities to bacteria likely represent annotation deficiencies rather than bacterial contamination. These data strongly indicate that by analysis of assembled contigs, we gain a more comprehensive view of the constituents of viromes than is typically observed from analysis of virome reads.

**Fig. 2:**
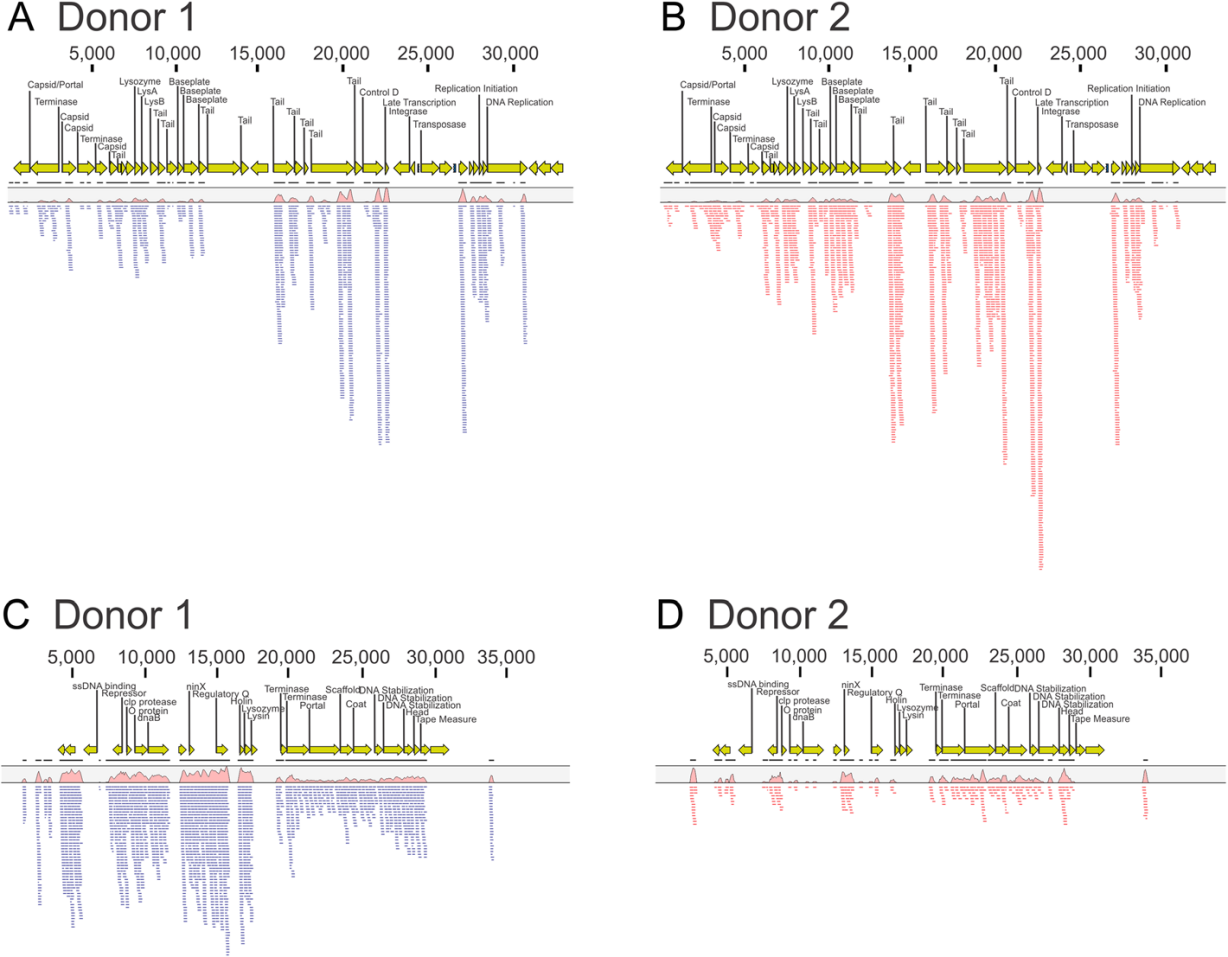
Read mappings of chemostat viromes from day 24 to Enterobacteria phage FIAA91ss (**a** and **b**) and Enterobacteria phage IME10 (**c** and **d**). **a** and **c** represent donor #1 and **b** and **d** represent donor #2. The genes and their respective directions are shown by the *yellow arrows*, and the annotation of each gene is represented above. The relative location along the phage genomes are demonstrated by the scale at the top of the diagram, and the relative proportion of reads mapping to the phages are shown at the lower portion of the diagrams

To decipher whether there may be viruses present in the cultured viral communities similar to those present in other public databases, we mapped the reads from each subject against a composite database of known viruses including the NCBI viral database and the Phantome database. We found that there were numerous different viruses that were matched by reads from the cultured viral communities. For example, many reads from day 24 mapped to Enterobacteria Phage FIAA91ss, including 1280 reads (0.14 % of the reads) from donor #1 (Fig. 2a) and 1981 reads (0.25 % of the reads) from donor #2 (Fig. 2b). Very few reads matching Phage FIAA91ss were found in donor #1 on days 4, 8, 12, and 16 (Additional file 3: Figure S2, Panel A). Similar results were found for donor #2; however, a higher number of reads were identified on day 12 and their proportion increased up to day 24 (Additional file 3: Figure S2, Panel B). Enterobacteria Phage FIAA91ss is a myovirus, and although many myoviruses lead primarily lytic lifestyles, the presence of predicted integrase and transposase genes in the FIAA91ss genome suggests that like other P2-like phages, it has a primarily lysogenic lifestyle. There also were many reads from day 24 that mapped specifically to Enterobacteria Phage IME10, including 985 reads (0.11 % of the reads) from donor #1 (Fig. 2c) and 255 reads (0.03 % of the reads) from donor #2 (Fig. 2d). Unlike Phage FIAA91ss, Phage IME10 was found in both donors at all time points (Additional file 3: Figure S3). Enterobacteria Phage IME10 is a podovirus, a group which generally lead lytic lifestyles; however, Phage IME10 has a predicted repressor protein that suggests it may be lysogenic. Many reads from both donors also mapped to Enterobacteria Phage HK620 at all time points, suggesting that a similar phage is present in the cultured viromes of both donors (Additional file 3: Figure S4). Interestingly, reads matching Enterobacteria phages FIAA91ss, IME10, and HK620 were identified in donors #1 and #2 (husband and wife) but were not identified in donors #10 (daughter), #8, or #9.

We also analyzed the cultured viral communities using the MG-RAST Server [43] and categorized those with sequences similar to known phages according to their families. We found that in all five donors, their cultured communities at all time points had reads similar to known myoviruses, podoviruses, and siphoviruses (Additional file 3: Figure S5). The proportions of reads similar to those different phage families generally differed substantially within a subject over time and between different subjects. The relative proportions of phage families observed do not necessarily reflect those found in the fecal viromes of each subject, particularly in donors #8 and #9. Viruses from the family Microviridae also were found in the feces of four of the five subjects, but none were identified in the chemostat cultures.

### Comparisons of fecal and cultured viral communities

We identified significant sequence similarities to each assembled viral contig by BLASTX analysis against the NCBI non-redundant database to decipher which viral genes had similarities in the chemostat cultures. The vast majority of the contigs had similarities to hypothetical phage proteins, proteins involved in replication/integration, restriction/modification enzymes, or tail fibers (Additional file 3: Figure S6). There were no significant differences identified in the relative proportions of contigs similar to different phage categories for either fecal or cultured phage communities regardless of the time point examined.

We previously demonstrated that phages in the mouth are highly persistent members of human oral microbial communities [10]. We utilized similar techniques to decipher whether phages in the chemostat cultures might also persist over time. By creating viral assemblies from all time points within a donor combined, we then could assess which different time points contributed to each assembly. We found that 45 ± 15 % of assemblies from all subjects included contigs from day 4, 42 ± 8 % from day 8, 44 ± 10 % from day 12, 38 ± 17 % from day 16, 37 ± 18 % from day 24, and 19 ± 7 % from feces (Additional file 3: Figures S7 and S8). The substantial difference in the percentage of assemblies that included fecal contigs suggests that there is less conservation in the phages present in feces compared to chemostat cultures. We also used BLASTN to compare the contigs between all donors and time points studied and found a similar pattern of conserved viruses over time in the chemostat cultures (Additional file 3: Figure S9). There was generally less conservation when comparing the chemostat culture viromes with those of the feces. The patterns of similar viruses across all donors suggested that there were individual-specific features of the viromes in each donor, giving the heatmap a “matrix-like” appearance. There was considerable similarity amongst the chemostat and fecal viromes of donors #2 and #10 (mother and child). To verify that the patterns of shared viruses we observed in the heatmap (Additional file 3: Figure S9) and the assemblies (Additional file 3: Figure S7) were statistically significant, we utilized a permutation test [44] to compare the proportions of shared viruses by sample type. For the cultured communities, 25 % of the virus contigs sampled had significant sequence similarities across all subjects compared with only 9 % when comparing cultured communities with fecal communities (Table 1), a difference that was close to statistical significance (*p* = 0.057). Similar results were found for fecal communities, where 25 % of the viruses sampled across subjects had significant sequence similarities compared with only 13 % when comparing between cultured communities and fecal communities (*p* = 0.141).

**Table 1:** Viral homologues within and between subject groups

	Virome		
	Percent similar within group^a^	Percent similar between groups^a^	*p* value^b^
Feces	25.04 ± 12.81	8.69 ± 5.44	0.0568
Chemostat	25.41 ± 10.23	13.15 ± 8.11	0.1410

^a^Based on the mean of 10,000 iterations. One thousand random contigs were sampled per iteration

^b^Empirical *p* value based on the fraction of times the estimated percent similar contigs for each group exceeded that between groups

**Fig. 3:**
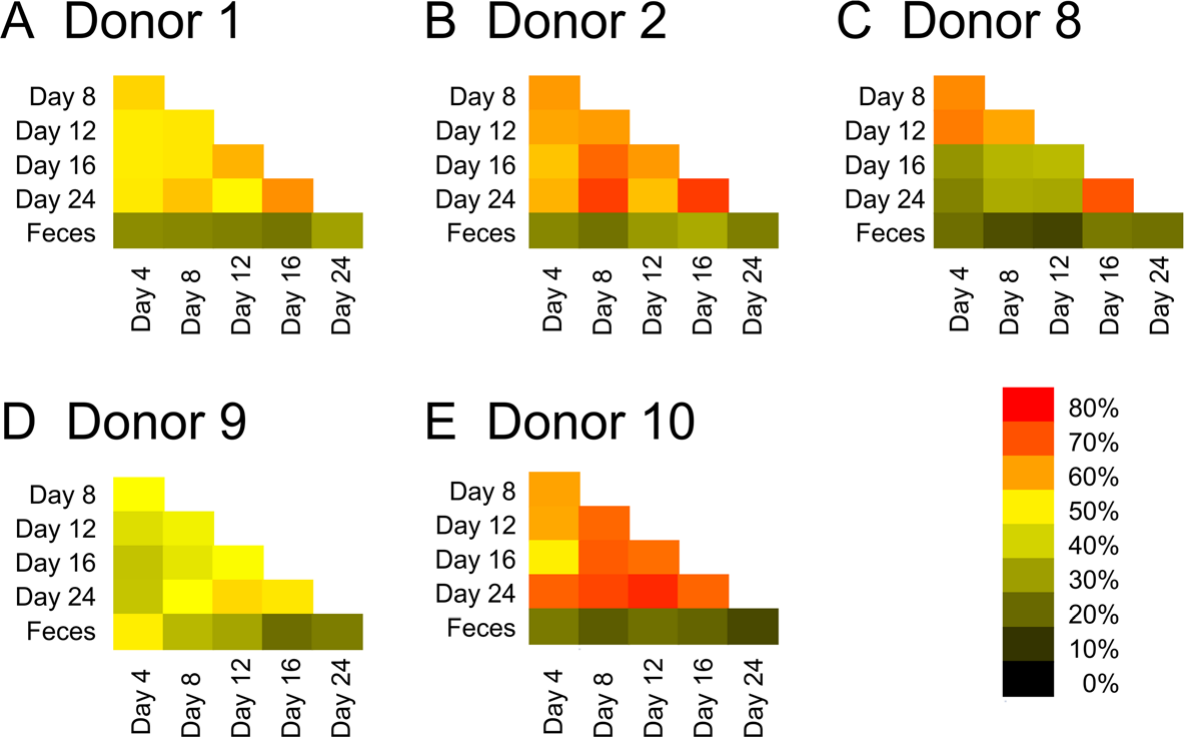
Heat matrices of the percentage of assemblies from each donor (**a-e**) that contained contigs from each time point and sample type

We created assemblies from all contigs in each donor to determine the relative proportions of viral contigs that were conserved in each donor over time. In donor #1, 53.3 ± 4.0 % of the contigs were conserved amongst the chemostat cultures over time compared to 25.9 ± 3.0 % conserved between the chemostat cultures and the feces (Fig. 3). In donor #2, 62.3 ± 6.2 % were conserved in chemostats compared to 26.5 ± 4.2 % between feces and chemostats; in donor #8, 44.3 ± 17.5 % compared to 18.6 ± 4.4 %; in donor #9, 45.4 ± 5.5 % compared to 32.2 ± 11.6 %; and in donor #10, 65.3 ± 7.1 % compared to 18.9 ± 3.5 %. In the chemostat cultures, the greatest conservation generally was between days 16 and 24, with 64.6 ± 8.4 % of the viral contigs conserved. These data indicate that while there are shared viruses between the chemostat cultures and the feces, there are identifiable differences in viral ecology between the sample types. Because our data also suggested that there were individual-specific features of each cultured virome, we used a permutation test to verify that the viral communities were significantly individual-specific across all time points in the cultured communities (Table 2). In all subjects studied, there was a statistically significant (*p* ≤ 0.05) trend observed, where the viruses in each of the cultured communities were significantly individual-specific.

**Table 2:** Chemostat and fecal virome homologues within and between subjects

	Virome		
	Percent similar within subject^a^	Percent similar between subjects^a^	*p* value^b^
Donor 1	54.38 ± 15.58	12.00 ± 5.26	<0.0001
Donor 2	47.07 ± 14.63	9.38 ± 5.47	=0.0012
Donor 8	44.01 ± 15.82	11.76 ± 6.26	=0.0068
Donor 9	50.43 ± 12.84	8.59 ± 3.70	<0.0001
Donor 10	54.19 ± 24.04	12.09 ± 8.85	=0.0374

^a^Based on the mean of 10,000 iterations. One thousand random contigs were sampled per iteration

^b^Empirical *p* value based on the fraction of times the estimated percent similar contigs for each group exceeded that between group

**Fig. 4:**
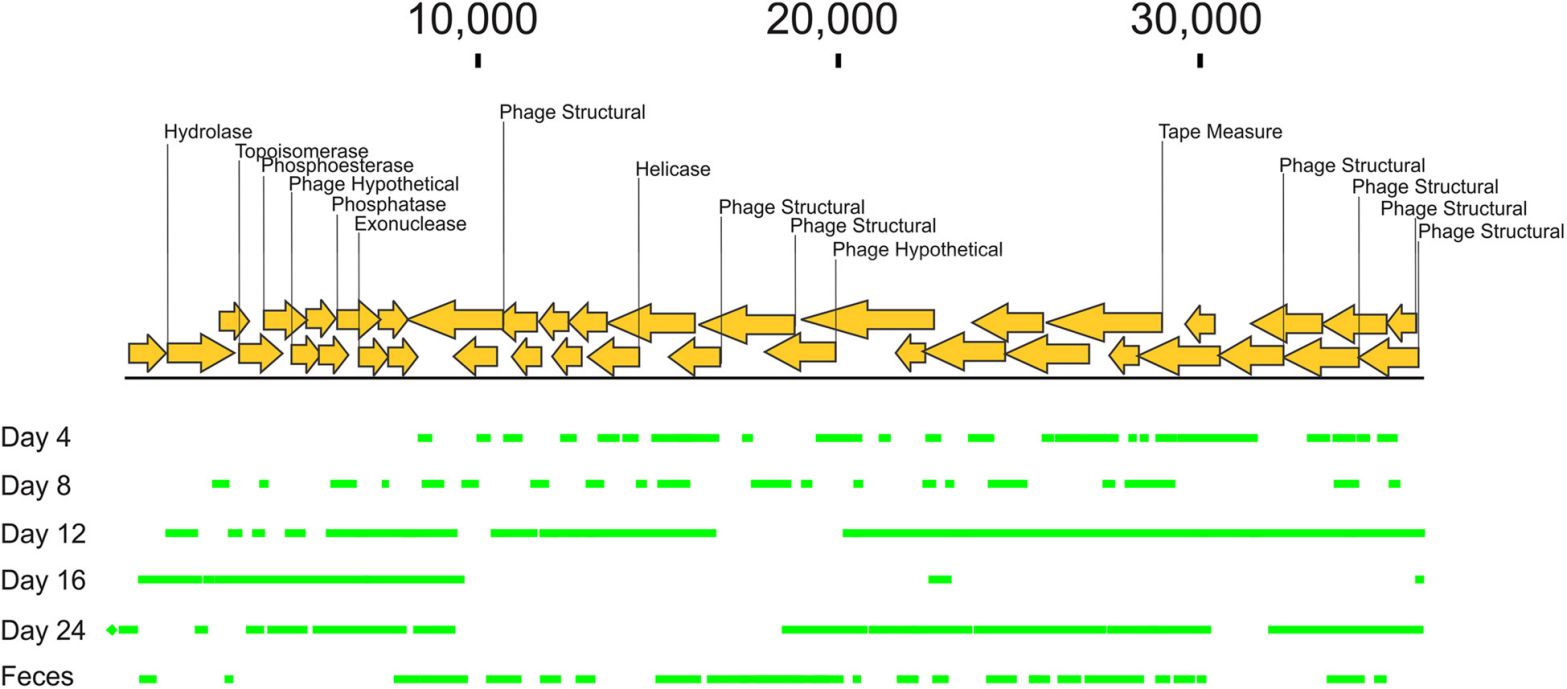
Assembly of contig 126 from all time points in donor #9. The portions of the contig identified in each time point or the feces are represented by the *colored boxes*. Putative ORFs and their directions are represented by the *yellow arrows*, and their annotations are represented above. The length of the contig is denoted at the top

We identified thousands of assemblies from all donors constructed from many different time points (Additional file 3: Figures S7 and S8). Each of these assemblies had identifiable phage sequence similarities. For example, in donor #9, we identified a 36,261 bp contig with numerous sequence similarities to phage genes across its length including hydrolase, helicase, and tape measure genes (Fig. 4). Similar results could be found for all donors; however, not all time points contributed equally to each assembly (Additional file 3: Figures S10, S11, S12 and S13). Interestingly, many of the assembled viruses had identifiable restriction/modification genes, which corroborate our findings of a high number of contigs with significant similarities to restriction/modification enzymes in the chemostat viromes (Additional file 3: Figure S6). M23 peptidases (Additional file 3: Figure S10), toxin-antitoxin genes (Additional file 3: Figure S11), S-layer, and platelet-binding proteins (Additional file 3: Figure S12) all had similar sequences identified in the phage genomes. A phage from donor #8 shared some synteny with crAssphage [45] (Additional file 3: Figure S13).

**Fig. 5:**
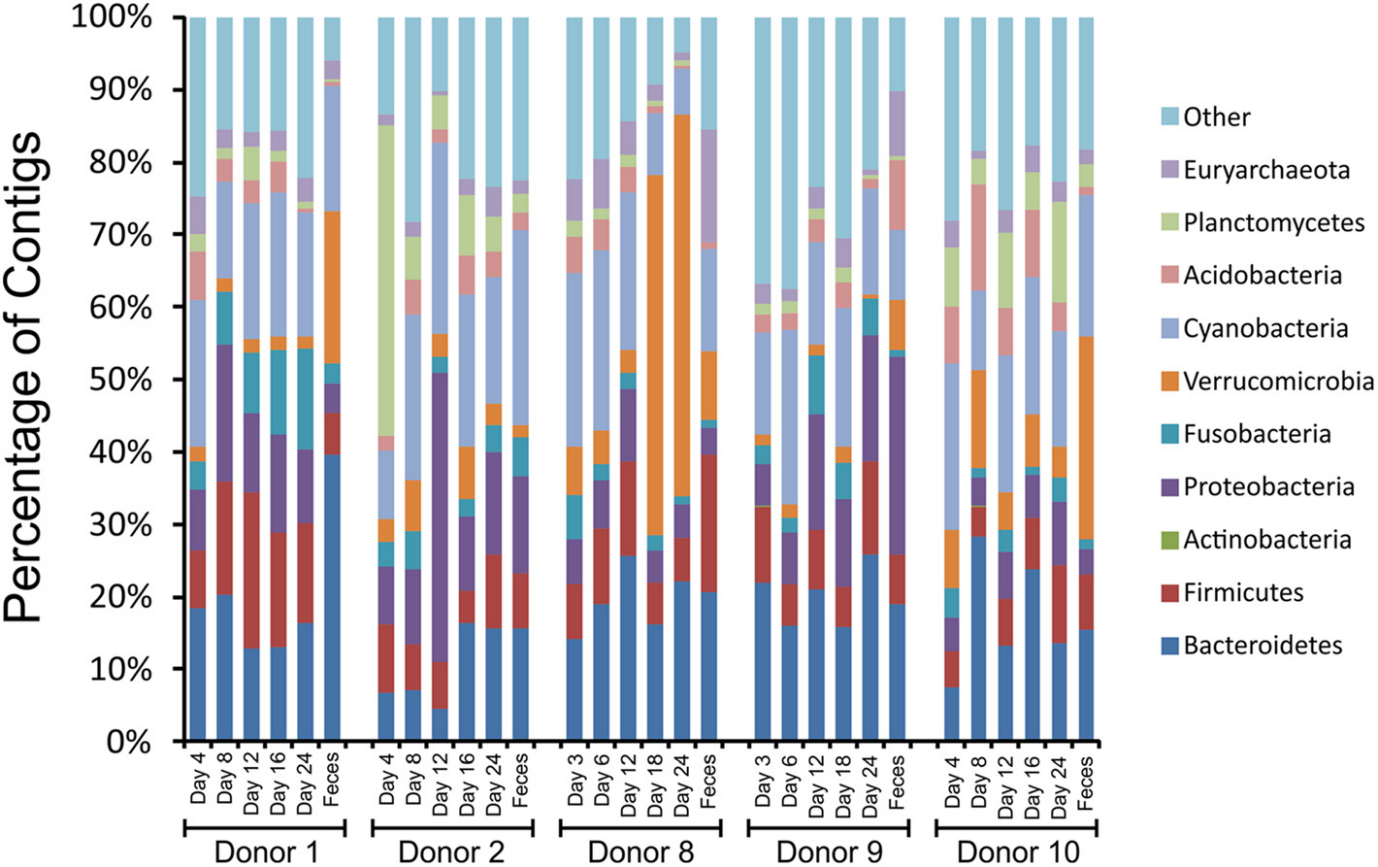
Bar graphs demonstrating proportion of contigs with BLASTX significant sequence similarities to phages that parasitize the specified bacterial phyla for fecal and chemostat viromes. *Each bar* from left to right represents the day of culture, and the *last bar* for each donor represents fecal viromes

We utilized the taxonomic information from the virome BLASTX hits to determine whether the phages from fecal and cultured communities had similar profiles. We found that the profiles of BLASTX hits differed between the different donors and varied based on the time point examined (Fig. 5). Within each donor, the profiles were somewhat similar over time with the most substantial profile differences between the chemostat cultures and the feces in most subjects. The most abundant phyla identified were Bacteroidetes and Proteobacteria, but Firmicutes, Fusobacteria, and Verrucomicrobia also were identified. There was a relatively high number of Verrucomicrobia identified in donors 8 and 10, which represented the genus Akkermansia. For comparison, we characterized the taxonomy of the bacterial communities in fecal and cultured communities using 16S rRNA genes. As expected, operational taxonomic units (OTUs) belonging to phyla Bacteroidetes and Firmicutes were amongst the most highly abundant taxa identified (Additional file 3: Figure S14).

**Fig. 6:**
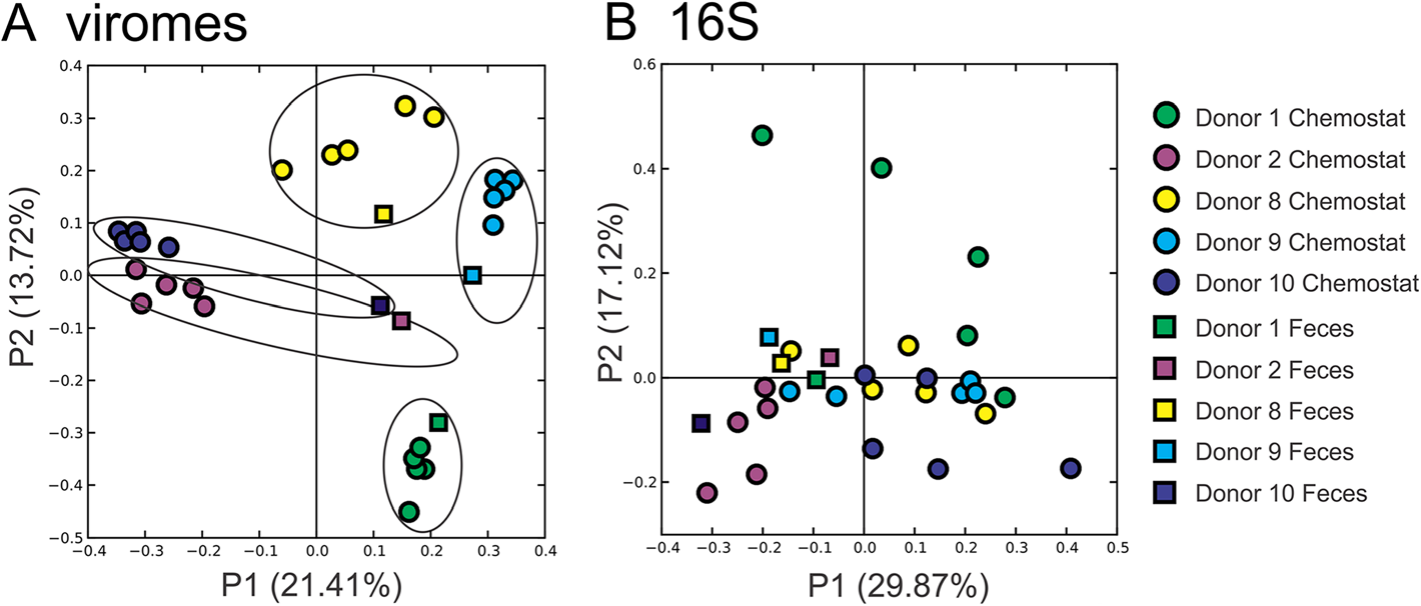
Principal coordinates analysis of beta diversity present in the viromes based on patterns of similar contigs between each virome (**a**) and bacteria by 16S rRNA genes (**b**) of each subject and sample type. Fecal samples are represented by *squares*, and chemostat viromes are represented by *circles*. In **a**, ovals are drawn around the data points for each individual donor

We utilized principal coordinates analysis (PCOA) based on the beta diversity between the fecal and cultured viromes to determine if there were patterns of variation specific to each subject and sample type. Each of the cultured viromes reflected the subject from which they were derived (Fig. 6a). While the variation present in fecal viromes could be distinguished from the cultured viromes, they clustered near the cultured viromes in each donor, indicating that there were shared features between the fecal and cultured viromes. The patterns of variation observed on PCOA were highly robust, as similar patterns were observed when the PCOAs were constructed based on contigs contributing to assemblies (Additional file 3: Figure S15A) and BLASTX hit profiles (Figure S15B). The distinction between the different donors was not as apparent when examining the bacteria using 16S rRNA genes (Fig. 6b). These data indicate that much of the individual-specific character of human fecal viromes was captured in chemostat culture systems.

### Viral diversity in fecal and cultured communities

**Fig. 7:**
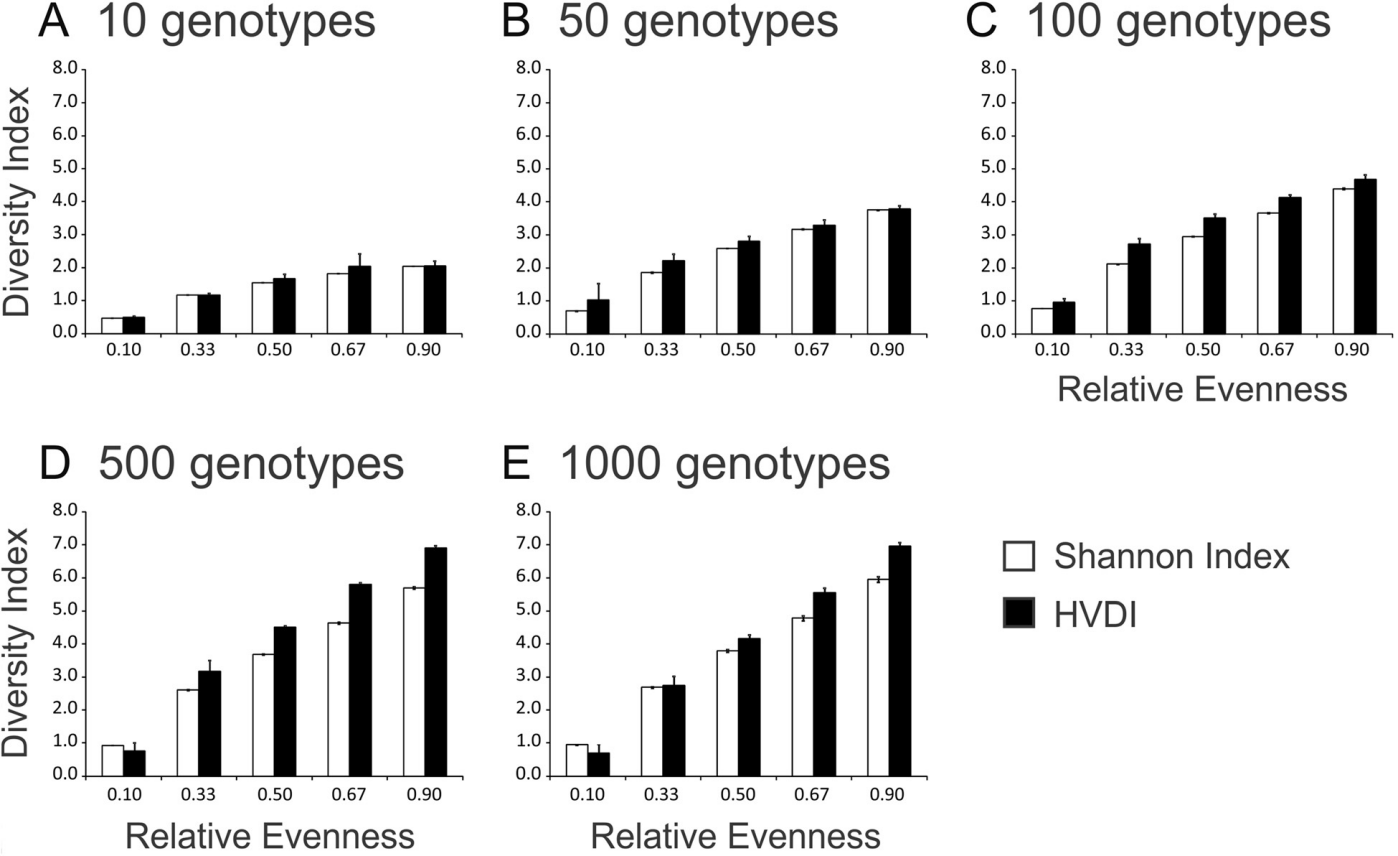
Bar graphs (±standard deviation) representing the Homologous Virus Diversity Index (HVDI) and Shannon Index values for a group of randomly constructed viromes. Each virome was constructed by randomly sampling amongst the viruses present in the NCBI and Phantome databases, and each was constructed to meet specific evenness values. The Shannon index was determined based on the actual sampling of the viruses in the databases, and the HVDI was determined after assembly of the randomly constructed viromes. For each evenness value, 10 separate iterations were performed on different sets of randomly sampled genomes. The y-axis represents values for either the Shannon Index or the HVDI, and the x-axis represents the evenness value to which the viromes were constructed to meet. **a**-**e** represent the different numbers of virus genotypes that were sampled

We developed tools for characterizing viral communities to discern whether richness and diversity were conserved between fecal and cultured communities. The technique, termed the Homologous Virus Diversity Index (HVDI), is primarily based upon the Shannon Index [46] and is used for comparing diversity between different communities. The HVDI utilizes contig spectra as a surrogate for population structures and corrects for the limitations imposed on the contig spectra by assembly methods by assigning the spectra for highly similar contigs to the same viral genotypes [47]. We validated the technique by randomly constructing viromes using viruses from the NCBI virus and Phantome databases to meet specific genotype and evenness requirements. Viromes were constructed with 10, 50, 100, 500, and 1000 different viral genotypes across an evenness spectrum consisting of 0.10, 0.33, 0.50, 0.67, and 0.90. We created 10 separate viromes at each genotype and evenness value to ensure the data were reproducible and used the number of genotypes and randomly sampled reads from each virome to calculate the Shannon Index (Fig. 7). We then used the contig spectra from the assembled reads from each virome as input for the HVDI. When only 10 different viruses were evaluated, the HVDI values approximate the Shannon Index across all evenness levels (Fig. 7a), and similar results were found for 50 viral genotypes (Fig. 7b). At higher numbers of genotypes (100, 500, and 1000), the HVDI exceeded the Shannon Index, although not considerably (Fig. 7c–e). The extent of the diversity overestimates were related to the evenness in the viromes, where the lowest evenness value of 0.1 resulted in the highest overestimates of diversity (Additional file 3: Figure S16). For evenness values of 0.33–0.9, the percentage differences between the HVDI and the Shannon Index were 12 % or lower and were highly consistent across the spectrum. These data indicate that the HVDI provided estimates of viral diversity similar to those of the Shannon Index and demonstrated that overestimates of viral diversity by the HVDI across different evenness levels were consistent and predictable.

We next used the HVDI to perform rarefaction analysis to determine whether the viruses in the viromes had been adequately sampled and as a measure of whether the richness of viruses differed substantially between fecal and cultured communities. In this case, we calculated the HVDI based on the Chao1 index [48] because it penalizes more heavily for the presence of the rarer viral contigs in each sample. We found that there was no association between the sample type and the richness within the viral communities and that the diversity estimates approached asymptote in many cases, indicating that little additional viral diversity would have been identified through further sampling (Additional file 3: Figure S17).

**Fig. 8:**
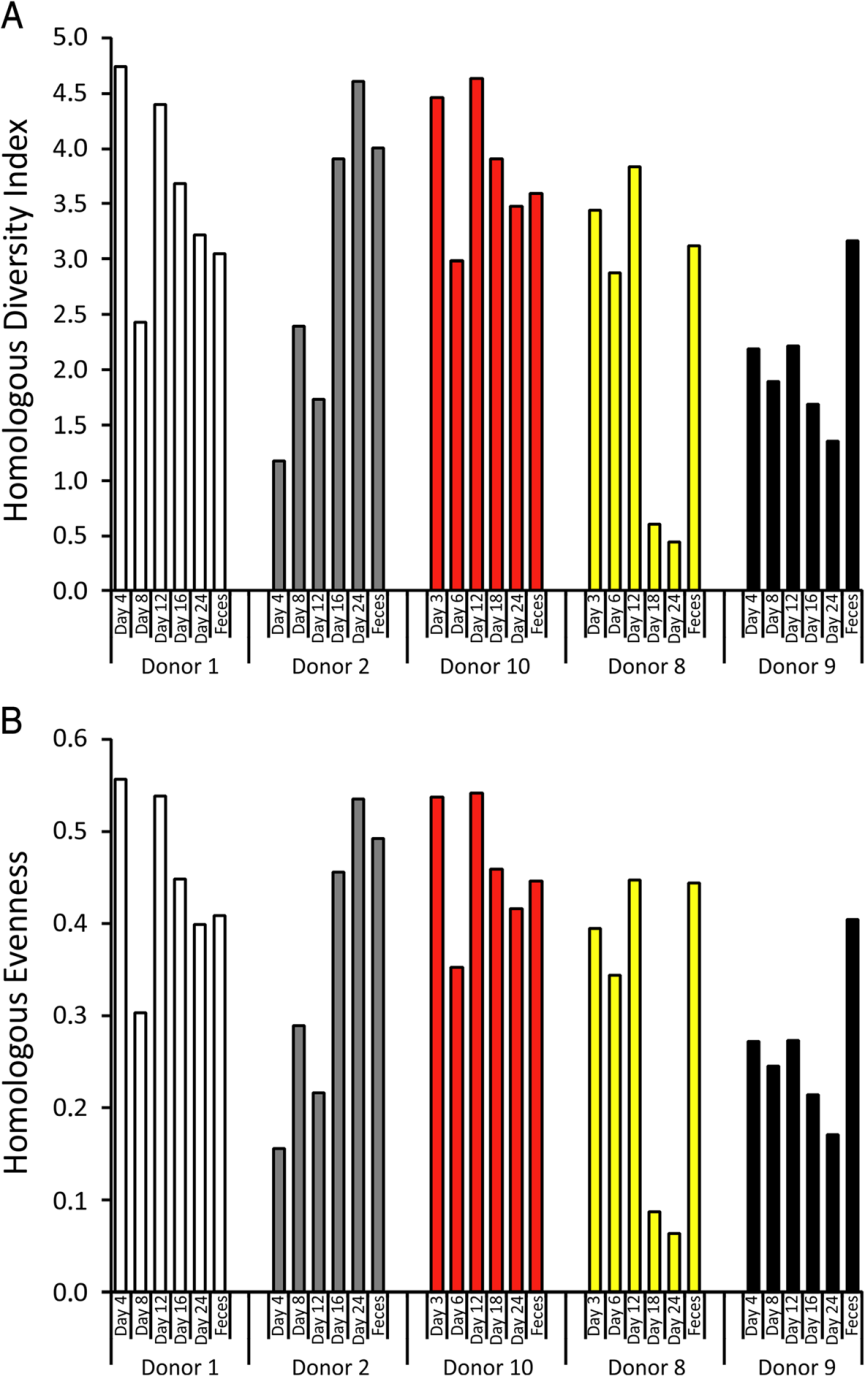
Bar graphs representing the Homologous Virus Diversity Index (**a**) and Homologous Evenness Index (**b**) for all subjects. The *y-axis* represents diversity, and on the x-axis *each bar* from left to right represents day of culture. The *last bar* for each donor represents the fecal viromes

We next compared the results of the HVDI using the Shannon Index to investigate whether the diversity of the viral component of cultured communities was similar to that from the feces in each subject. We found that for all subjects, viral diversity in the cultured communities changed as a function of time. For example, in donor #2, viral diversity generally increased from day 4 to day 24, while viral community diversity generally decreased in donor #9. For donors #1, #2, and #10, the diversity present in cultured communities on day 24 was highly similar to that in the feces of each subject (Fig. 8a). For donors #8 and #9, the diversity in cultured communities diminished significantly by day 18 which continued through day 24. Neither of the day 24 viromes in donors #8 or #9 approximated the diversity present in the fecal viral communities. Evenness values for all subjects and time points were highly consistent with the HVDI data (Fig. 8b). Most of the evenness estimates ranged from 0.2 to 0.6, which represents the range which the HVDI likely is to overestimate diversity by approximately 12 % (Additional file 3: Figure S16). The evenness values for viral communities in donors #8 and #9 diminished significantly, which suggests that these communities were populated by fewer viruses that represented the majorities of the population. We repeated the virome preparations from the cultured communities on days 24 from donors #8 and #9 and similar results to those observed were found, indicating that there likely was a significant drop in viral diversity in these chemostat cultures (data not shown). Interestingly, the number of FSPs was not different for these cultured communities in donors #8 and #9, so the differences in evenness cannot be attributed to fewer total viruses being present. Alpha diversity for the bacteria as determined by the Shannon Index increased from day 4 to 24 and more closely approximated the feces by day 24 (Additional file 3: Figure S18). These data suggest that the relative loss of viral diversity in the chemostat cultures was not due an overall loss of bacterial diversity.

## Discussion

Human body surfaces are home to large populations of viruses, whose role as members of human microbial communities is not well understood. In some environments, viral communities have been shown to be involved in driving bacterial diversity, yet no such data exists for human viral communities. We know that many of the phages that populate these communities are highly persistent and carry numerous pathogenic gene functions such as antibiotic resistance, which could help to shape bacterial community membership and its response to certain perturbations. Measuring their effects in humans is not without its challenges, which include the need to use drugs such as antibiotics, the need to reduce confounders such as diet variability, and the need to improve compliance with study protocols. The development of ecosystems that can approximate the dynamic interactions between phage communities and their cellular hosts can greatly reduce the reliance on human subjects in the characterization of human microbial communities.

Each virome in this study was subjected to MDA amplification due to the small amounts of viral DNA recovered from each donor. MDA amplification is known to introduce biases into sequence data [49], and it is unclear how MDA amplification biases could have affected these viromes. Many of the biases introduced often result when relatively low levels of starting DNA are utilized for amplification, which could have occurred in some of the viromes in this study. The relative conservation of viral genotypes across different time points in the chemostat cultures (Fig. 3) suggests that if MDA amplification biases affected the analysis of the viromes, the effects may have been relatively uniform across time points. The relative abundances of Microviridae, however, in the feces of four of the five subjects, may have been overrepresented through the use of MDA, as has previously been described [49]. The absence of microviruses in the chemostat cultures suggests that their bacterial host species may not be well represented in chemostat cultures.

Different bacteriophage families often have different lifestyles. Because phages can be significant drivers of diversity in different ecosystems [3-5, 11, 12, 39, 50], we examined whether there were phages with significant sequence similarities to known phage families in our cultured viral communities. Caudoviruses are the phage families most often found in our prior analyses of human oral viral communities [9, 10] and were commonly identified in the viral cultures in this study (Additional file 3: Figure S5). Of the different types of caudoviruses, siphoviruses generally have lysogenic lifestyles, while podoviruses and myoviruses more often have lytic lifestyles. We identified similar sequences to each of these viral families in our analysis of cultured viral communities, which strongly suggests that both primarily lytic and lysogenic phages were present. Not all myoviruses and podoviruses, however, have lytic lifestyles, as demonstrated by the presence of Enterobacteria phages FIAA91ss, IME10 (Fig. 2), and HK620 (Additional file 3: Figure S4), which have predicted gene functions that indicate their probable lysogenic lifestyles. The presence of each of these caudovirus families indicates that each of these virus types is viable in cultured communities. We found reads matching Enterobacteria phages FIAA91ss, IME10, and HK620 in the husband and wife but not in their daughter or the other donors. This suggests that these viruses were transmitted between husband and wife, but not to their offspring, potentially due to a lack of suitable host bacteria in the daughter.

Because of the high diversity in human phage communities and the varying relationships between host and phages, the relative abundances of phages do not necessarily reflect those of their host bacteria. This phenomenon was demonstrated in a prior study of human oral viral communities [39] and in a more recent study in the guts of humans with inflammatory bowel disease [51]. The data in this study support this finding, as the relative abundances of phage BLASTX hits from chemostat cultures and feces (Fig. 5) were not reflective of the relative abundances of the bacteria (Additional file 3: Figure S14). While BLASTX hit profiles do not necessarily represent taxonomic classifications, the abundances of phylum Bacteroidetes found in the phage BLASTX profiles and the 16S rRNA gene profiles suggest that many phages parasitizing these bacteria were present in the feces and chemostat cultures. Few Proteobacteria were identified through analysis of 16S rRNA genes (Additional file 3: Figure S14), yet the abundance of phage hits to Proteobacteria was high (Fig. 5). These results may have been influenced by an unequal representation of phage from Proteobacteria in available databases, where a high relative abundance of phage from Proteobacteria renders us more likely to identify hits to Proteobacteria even when Proteobacteria are not the hosts of these phage.

Our prior studies of human viral communities have demonstrated that diversity in these communities is generally overestimated [10]. In that study, we found numerous different contigs that could not assemble, which actually belonged to the same phages. We developed a method to reduce the overestimation of viral diversity by finding high levels of similarity amongst these contigs and assigning their contig spectra to the same virus genotype. We do not believe that in every case high levels of similarity necessarily represent the same virus; however, utilizing this technique allowed us to estimate viral community diversity within 12 % of the actual diversity in our simulated communities across most evenness levels. This technique allowed for us to provide estimates of sequencing depth adequacy as well as comparisons of diversity amongst fecal and cultured viral communities. While the data indicate that most of the viral diversity in these communities could be identified by sequencing <20,000 reads (Additional file 3: Figure S15), the greater number of reads used in this study was necessary to assemble more reliable contig spectra. The rarefaction analyses strongly suggested that further sequencing would not have added substantially to the diversity estimates present in cultured or fecal viral communities. We believe that tools such as the HVDI add to available methodologies for examining viral community diversity and will be of great utility in understanding the responses of viral communities to perturbations.

One of the major goals of this work was to examine how closely cultured communities might approximate human indigenous phage communities. Identifying numerous FSPs and many reconstructed phages in the cultured communities (Figs. 2 and 4, and Additional file 3: Figures S10, S11, S12 and S13) strongly suggests that chemostat culture systems are fully capable of supporting robust phage communities. Chemostat cultured communities have been known to support the viability of individual phages [30]; however, the data presented here indicate that chemostat cultures can support entire communities of phages as well. Our focus on contigs rather than virome reads allowed us to characterize 94.9 % of the viromes (far greater than often is reported in virome studies), which we believe provides a broad overview of the phage present in chemostat cultures and feces. The significant drop in diversity and evenness in the viromes in donors #8 and #9 by day 18 cannot be attributed to a drop in bacterial diversity (Additional file 3: Figure S14), and the BLASTX profiles in these subjects (Fig. 5) suggest that while diversity was diminished, it likely was diminished across different bacterial host lineages. While these cultured communities do not perfectly approximate the diversity of phages in the human gut, as evidenced by the generally higher similarities within chemostat viromes compared with fecal viromes (Fig. 3), the ability to approximate diversity in some human viromes (Fig. 8) should prove useful for furthering our understanding of host/phage interactions in humans. We did not perform detailed comparisons of phages and their putative bacterial hosts in each donor because BLASTX is generally considered insufficient to accurately predict the hosts of each phage.

## Conclusions

By establishing phage communities that have some similarities to those found on human body surfaces, several important questions can be addressed. These questions include the following: (1) what is the role of phages in driving the diversity of the bacteria in human microbial communities?, (2) how do perturbations such as antibiotics impact human phage communities?, (3) how does the sharing of phage communities between individuals impact microbial community membership?, and (4) what are the dynamics of most phages as members of human microbial communities? While there are quantifiable differences between the phage communities present in feces and cultured communities, there also are many similarities. The relative number of FSPs in both sample types are similar, the profiles of beta diversity strongly suggest a conservation of some phage community members across fecal and cultured communities, and the relative levels of phage diversity between communities in some subjects were highly similar. By establishing cultured phage communities, we can begin to understand the role and contributions of phages to human microbial communities.

## Methods

### Ethics, consent, and permissions

Human subject recruitment and enrollment in this study was approved by The Research Ethics Board of the University of Guelph REB#13AP008 and 10JL002. Each subject signed a written informed consent confirming their willingness to participate in this study.

### Human subjects

Five healthy donors provided fecal samples: donor #1 (male, 44 years old), donor #2 (female, 41 years old), donor #8 (female, 26 years old), donor #9 (male, 25 years old), and donor #10 (female, 7 years old). Donors #1, #2, and #10 were a co-habiting family unit of father, mother, and child, respectively. Donors #8 and #9 were unrelated. None of the donors had a recent history of antibiotic treatment for 9 months prior to sampling. Each donor provided at least 5 g of fresh fecal samples for the chemostat cultures. Donor #10 provided a sample in the home environment which was immediately wrapped in plastic cling wrap to exclude air and then frozen at −20 °C for overnight transportation to the lab. The remaining donors provided fresh samples.

### Chemostat cultures

All samples were placed immediately into an anaerobic chamber (90 % N_2_, 5 % CO_2_, 5 % H_2_) upon receipt at the lab; for the fresh samples, this was within 30 min of collection. The sample from donor #10 was allowed to thaw in the chamber. A modified Infors Multifors system was used to run chemostat cultures modeling the human distal colon environment, as described by McDonald et al. [29]. A 10 % (*w*/*v*) slurry was prepared from each donor by homogenizing feces in pre-reduced growth medium using a stomacher. For every 1 L of medium, the following components were included in the growth medium: peptone water, 2 g; yeast extract, 2 g, NaHCO3, 2 g; CaCl2, 0.01 g; pectin (from citrus), 2 g; xylan (from beechwood), 2 g; arabinogalactan, 2 g; starch (from wheat, unmodified), 5 g; casein, 3 g; inulin (from Dahlia tubers), 1 g; bile salts, 0.5 g NaCl, 0.1 g; L-cysteine HCl, 0.5 g; K2HPO4, 0.04 g; KH2PO4, 0.04 g; MgSO4, 0.01 g; hemin, 0.005 g; and menadione, 0.001 g (all components from Sigma Aldrich). Growth media was stored at 4 °C until use for a maximum of 2 weeks. The fecal slurry in growth medium was then centrifuged to remove large particles [57], and the supernatant was used as the inoculum for each experiment by adding 100 mL into 300 mL of sterile growth medium in each vessel. The pH within each vessel was then adjusted to 6.9–7.0, and the cultures gently and continually agitated and maintained at 37 °C. Vessels were run in batch mode for 24 h to allow inoculum recovery time (adjustment from in vivo to in vitro conditions) and to avoid wash-out. The pumps were then switched on, and the retention rate set to 16.67 mL/h^−1^ with constant sparging of O_2_ free N_2_ gas to maintain positive pressure and anaerobiosis. Each chemostat vessel was sampled daily by aseptically removing 4 mL of culture directly from the vessel contents, and all samples were archived at −80 °C. Aliquots of the original fecal samples also were archived at −80 °C for virome processing.

### Preparation and sequencing of viromes

Fecal viromes were prepared by diluting 0.4 g of feces in 4 mL of SM buffer. The fecal samples were vortexed vigorously for 40 min to separate viral particles, spun at 4000×*g* for 10 min to pellet the remaining solid material, and the supernatant was treated in an identical manner to that of the chemostat cultures. A small portion (10 μL) of the supernatant from each donor was resuspended in 190 μL of 0.02-μm filtered PBS and their counts per milliliter determined by epifluorescence microscopy [38]. Chemostat samples and fecal supernatants were filtered sequentially using 0.45 and 0.2 μm filters (VWR, Radnor, PA) to remove cellular and other debris and then purified on a cesium chloride gradient according to previously described protocols [39]. Only the fraction with a density corresponding to most known bacteriophages [52] was retained, further purified on Amicon YM-100 protein purification columns (Millipore, Inc., Bellerica, MA), treated with DNase I, and subjected to lysis and DNA purification using the Qiagen UltraSens Virus kit (Qiagen, Valencia, CA). Recovered DNA was screened for the presence of contaminating bacterial nucleic acids by quantitative 16S rRNA gene PCR using primers 8F (AGAGTTTGATCCTGGCTCAG) and 357R (CTGCTGCCTYCCGTA) in Power SYBR Green PCR Mastermix (Thermo Fisher Scientific, Carlsbad, CA). No products were detected in any of the viromes after 35 cycles, which does not exclude the presence of contaminating bacterial nucleic acids but indicates that they were not present at dominant levels. Resulting DNA was amplified using GenomiPhi Hy MDA amplification (GE Healthcare, Pittsburgh, PA), fragmented to roughly 200 to 400 bp using a Bioruptor (Diagenode, Denville, NJ), and utilized as input to create libraries using the Ion Plus Fragment Library Kit according to manufacturer’s instructions. Libraries then were sequenced using 314 or 316 chips on an Ion Torrent Personal Genome Machine (PGM; Life Technologies, Grand Island, NY) [40] producing an average read length of approximately 215 bp for each sample.

### Analysis of viromes

Due to the error rate of semiconductor sequencing [40], we trimmed each read according to modified Phred scores of 0.5 using CLC Genomics Workbench 8.01 (CLC bio USA, Cambridge, MA), removed any low complexity reads with ≥8 consecutive homopolymers, and removed any reads with substantial length variation (<50 nucleotides or >300 nucleotides) or ambiguous characters prior to further analysis. Each virome was screened for contaminating bacterial and human nucleic acids using BLASTN analysis (E-value <10^−5^) against the Ribosomal Database Project 16S rRNA genes database [53] and the human reference database available at ftp://ftp.ncbi.nlm.nih.gov/genomes/H_sapiens/. Any reads with significant sequence similarities to human sequences were removed prior to further analysis. Length and GC content variation amongst contigs was assessed using Box and Whiskers plots created using Microsoft Excel 2007 (Microsoft Corp., Redman, WA). All reads were assembled using CLC Genomics Workbench 8.01 based on 98 % identity with a minimum of 50 % read overlap, which were more stringent than criteria developed to discriminate between highly related viruses [54]. The assembly method works by using a de Bruijn graph technique and various word lengths, similar to that used in the assembler Velvet [55]. We also used MetaVelvet [41] and IDBA-UD [42] in the construction of contigs, but the CLC assembler produced fewer contigs of higher mean lengths with greater N50 values (Additional file 2: Table S2). Because the shortest reads were 50 nucleotides, the minimum tolerable overlap was 25 nucleotides, and the average overlap was no less than 100 nucleotides depending on the characteristics of each virome. The consensus sequence for each contig was constructed according to majority rule, and any contigs <200 nucleotides or with ambiguous characters were removed prior to further analysis. Read mapping of viromes to a combined database of viruses (www.phantome.org; ftp://ftp.ncbi.nih.gov/genomes/Viruses/) was performed using CLC Genomics Workbench 8.01 (CLC bio USA, Cambridge, MA) and were mapped using 98 % identity over a minimum of 50 % of the read length. For each donor, we also utilized a separate technique for assembly by constructing global assemblies from all contigs from all time points using 98 % identity over a minimum of 50 % overlap. Viral contigs were analyzed using FGenesV (Softberry Inc, Mount Kisco, NY) for ORF prediction and individual ORFs analyzed using BLASTP analysis against the NCBI non-redundant database (E-value <10^−5^). If the best hit was to a gene with no known function, lower level hits were used for the annotation as long as they had known putative function and still met the E-value cutoff (10^−5^).

Contigs were annotated using BLASTX against the NCBI NR database with an E-value cutoff value of 10^−5^. Specific viral sequences were identified by parsing BLASTX results for known viral genes including replication, structural, transposition, restriction/modification, hypothetical, and other genes previously found in viruses for which the E-value was at least 10^−5^. Each individual virome contig was annotated using this technique; however, if the best hit for any portion of the contig was to a gene with no known function, lower level hits were used as long as they had known function and still met the E-value cutoff. The annotation data were compiled by the number of reads used to assemble each contig for each subject and used to determine the relative proportions of contigs that contained viral sequences. The phyla of the BLASTX best hits for each annotated contig were used to create profiles in each donor and sample type. We utilized the average coverage in the assemblies of each contig to determine the relative abundance profiles of different phyla to compensate for viruses that may be more abundant than others. This technique prevented reads involved in the assembly of the same virus contigs from being assigned to different putative host phyla based on different BLASTX similarities. Determination of the relative abundances of virus families was determined by BLASTX analysis of the SEED database using MG-RAST [43].

Analysis of shared sequence similarities present in each virome was performed by creating custom BLAST databases for each virome, comparing each database with all other viromes using BLASTN analysis (E-value <10^−10^). Principal coordinates analysis (PCOA) was performed on virome contigs with Bray Curtis distances using Qiime [56]. We also utilized a separate technique for assembly by constructing global assemblies from all contigs from all subjects and time points using 98 % identity over a minimum of 50 % overlap. The contribution of each subject and time point to each assembly was assessed and utilized to determine relative persistence of phages over time in the chemostat cultures and as input for PCOA and for heat matrix analysis using Microsoft Excel. We also used the profiles of BLASTX best hits amongst all subjects and time points as input for PCOA. Beta diversity based on Bray Curtis distances was used as input for each PCOA.

### Analysis of 16S rRNA genes

Genomic DNA was prepared from the feces of each subject and time point using the Qiagen QIAamp DNA Stool Mini kit (Qiagen, Valencia, CA). We amplified the bacterial 16S rRNA gene V1-V2 hypervariable region using the forward primer 8F fused with the Ion Torrent Adaptor A sequence and one of 23 unique 10 base pair barcodes and reverse primer 357R fused with the Ion Torrent Adaptor P1 from the each donor and sample type [57]. PCR reactions were performed using Platinum PCR High Fidelity SuperMix (Invitrogen, Carlsbad, CA) with the following cycling parameters: 94 °C for 10 min, followed by 30 cycles of 94 °C for 30 s, 53 °C for 30 s, 72 °C for 30 s, and a final elongation step of 72 °C for 10 min. Resulting amplicons were purified on a 2 % agarose gel stained with SYBR Safe (Invitrogen, Carlsbad, CA) using the MinElute PCR Purification kit (Qiagen, Valencia, CA). Amplicons were further purified with Ampure XP beads (Beckman-Coulter, Brea, CA), and molar equivalents were determined for each sample using a Bioanalyzer 2100 HS DNA Kit (Agilent Technologies, Santa Clara, CA). Samples were pooled into equimolar proportions and sequenced on 314 chips using an Ion Torrent PGM according to manufacturer’s instructions (Life Technologies, Grand Island, NY) [40]. Resulting sequence reads were removed from the analysis if they were <180 nucleotides, had any barcode or primer errors, contained any ambiguous characters, or contained any stretch of >8 homopolymers. Sequences were assigned to their respective samples based on a 10-nucleotide barcode sequence and were analyzed further using the Qiime pipeline [56]. Briefly, representative OTUs from each set were chosen at a minimum sequence identity of 97 % using UClust [58] and aligned using PyNast [59] against the Greengenes database [60]. Multiple alignments then were used to create phylogenies using FastTree [61], and taxonomy was assigned to each OTU using the RDP classifier [62, 63]. PCOA was performed based on beta diversity using weighted Unifrac distances [64].

### Statistical analysis

To assess whether virome contigs had significant overlap within or between donors and sample types, we performed a permutation test based on resampling (10,000 iteration). We simulated the distribution of the fraction of virome sequences with significant similarities from two different sample types within a donor. For each set, we computed the summed fraction of sequences with significant similarities using 1000 random contigs between different donors, and from these computed an empirical null distribution of our statistic of interest (the fraction of shared similar sequences). The simulated statistics within each donor were referred to the null distribution of inter-donor comparisons, and the *p* value was computed as the fraction of times the simulated statistic for each exceeded the observed statistic. For analysis of fecal versus cultured viromes, a randomly chosen donor from the cultured virome group was compared with a randomly selected donor from the fecal group to determine the null distribution of the fraction of shared contigs. We then estimated the fraction of shared sequences with significant similarities from randomly chosen donors within the cultured virome group and compared with the empirical null distribution from simulated inter-group values. Intra-subject comparisons were excluded from this analysis. We estimated the *p* value based on the fraction of times the intra-group statistic exceeded that for the null statistic.

### Homologous virus diversity index and virome construction

To measure alpha diversity in the viral communities, we utilized a technique termed the Homologous Virus Diversity Index [47]. The technique is based on finding high levels of similarity amongst contigs within viromes that potentially belong to the same or a highly similar viral genotype but were placed into separate contigs due to the limitations of the assembly process [10]. We validated the technique by constructing viromes composed of randomly selected viruses amongst both the NCBI (ftp://ftp.ncbi.nlm.nih.gov/genomes/Viruses/) and Phantome viral databases (www.phantome.org). First, a random set of viruses was chosen from amongst the databases for each virome, and viromes were constructed that contained 10 viruses, 50 viruses, 100 viruses, 500 viruses, and 1000 viruses. We constructed 10 different viromes at each level of different viral genotypes. The reads in each virome were of mean length 200 bp and were chosen randomly across the genomes of each virus. The shortest reads were 150 bp, and the longest reads were 250 bp in each constructed virome. Each virome also was constructed to meet specific evenness requirements, where an evenness of close to 1 would indicate a population consisting of different viruses all of similar relative abundances, and an evenness close to 0 would indicate a population where the relative abundance of a few viruses are much greater than others. We utilized evenness as criteria to construct the viromes because higher evenness values would prove more difficult for the assembly process. For each constructed virome, we created contig spectra by counting the number of reads assigned to each different virus in the virome. The contig spectra then were used as surrogates for population structures in determination of the Shannon Index [46]. The actual evenness values for each constructed virome were determined using the equation H/ln(S), where H is the Shannon Index value and S is the total number of viruses in each virome. We also determined the HVDI for each constructed virome by assembling the reads using 98 % identity over a minimum of 50 % of the read length using CLC Genomics Workbench 8.01 (CLC bio USA, Cambridge, MA). The resulting contigs then were subjected to BLASTN analysis against a database of contigs from the exact same subject, and contigs with high degrees of similarity (E-value < 10^−20^) over 50 % of the length of the shorter contig were assigned to the same viral genotype. The spectra from each individual contig that were assigned to the same genotype were added to a corrected contig spectrum for each subject and those spectra used as inputs for the Shannon Index [46] to calculate the HVDI. The actual Shannon Index values for each constructed virome were compared to the results of the HVDI in each case, and the HVDI estimates exceeded the Shannon Index by approximately 12 % across most evenness levels. Rarefactions were determined for each donor and time point by randomly sampling up to 20,000 reads. The randomly sampled reads then were assigned to their respective contigs by comparing them with the corrected contig spectra generated after the BLASTN analysis. We then constructed new contig spectra based on the sampled reads and their assignments to different viral genotypes and used those spectra in calculations of the HVDI. For the rarefactions, we calculated the HVDI using the Chao1 index [48], which utilizes the relative numbers of viral contigs created from only a single read (singleton) or from only two reads (doubleton) as input.

## Availability of data and materials

All sequences including viromes and 16S rRNA genes are available for download in the MG-RAST database (metagenomics.anl.gov/) under the project “Chemostat,” or project #10563.

## Additional files

Additional file 1: Table S1. Chemostat and fecal virome reads. Additional file 2: Table S2. Virome read assembly metrics. Additional file 3:
**Supplemental figures.**
