# Supplementary material for: Chemostat culture systems support diverse bacteriophage communities from human feces
Source: Microbiome. 2015 Nov 9;3:58. doi: 10.1186/s40168-015-0124-3 (PMC4638026; doi:10.1186/s40168-015-0124-3)
Supplement: Additional file 1: Table S1. — Chemostat and fecal virome reads. [file 40168_2015_124_MOESM1_ESM.pdf]

Table S1: Chemostat and fecal virome reads

Sample		Initial Reads	Mean Length	16S	Human	Final Reads	Contigs	GC Content	Mean Length
Donor 1									
	Day 4	507,611	200	0	1,381	506,230	5,457	45.5	947
	Day 8	767,240	208	0	405	766,835	3,280	44.8	948
	Day 12	504,907	205	0	475	504,432	3,926	43.7	1,033
	Day 16	732,018	204	0	484	731,534	4,064	43.9	997
	Day 24	895,131	215	0	466	894,665	3,383	46.6	939
	Feces	334,278	221	0	6	334,272	1,879	40.7	829
Donor 2									
	Day 4	486,907	201	0	287	486,620	1,946	48.9	686
	Day 8	650,442	208	0	707	649,735	4,206	48.4	790
	Day 12	585,238	204	0	395	584,843	3,201	46.1	773
	Day 16	724,678	199	0	2,537	722,141	5,698	46.0	1,025
	Day 24	808,277	223	0	1,092	807,185	6,022	50.8	998
	Feces	615,197	227	0	1,503	613,694	3,739	42.5	1,014
Donor 8									
	Day 3	533,819	222	0	977	532,842	6,541	47.7	846
	Day 6	476,648	224	0	468	476,180	4,547	46.0	821
	Day 12	659,253	221	0	980	658,273	5,781	47.0	890
	Day 18	645,691	213	0	100	645,591	1,114	46.6	876
	Day 24	690,421	224	0	58	690,363	1,018	46.3	815
	Feces	686,334	218	0	808	685,526	1,266	44.3	1,103
Donor 9									
	Day 3	651,601	200	0	133	651,468	3,391	42.8	827
	Day 6	662,865	212	0	164	662,701	2,425	41.2	829
	Day 12	558,946	222	0	440	558,506	3,556	42.4	901
	Day 18	509,012	215	0	700	508,312	2,867	43.6	909
	Day 24	643,136	222	0	44	643,092	2,913	43.5	649
	Feces	463,689	224	0	80	463,689	2,696	40.0	924
Donor 10									
	Day 4	622,317	211	0	3,909	618,408	4,533	51.0	1,219
	Day 8	713,608	220	0	1,243	712,365	5,148	47.8	927
	Day 12	561,765	220	0	2,193	559,572	5,891	48.3	1,107
	Day 16	578,809	224	0	1,255	577,554	5,365	46.1	931
	Day 24	704,854	225	0	1,495	703,359	4,808	51.5	1,015
	Feces	634,880	227	0	263	634,617	3,364	41.1	956
