# Supplementary material for: Chemostat culture systems support diverse bacteriophage communities from human feces
Source: Microbiome. 2015 Nov 9;3:58. doi: 10.1186/s40168-015-0124-3 (PMC4638026; doi:10.1186/s40168-015-0124-3)
Supplement: Additional file 2: Table S2. — Virome read assembly metrics. [file 40168_2015_124_MOESM2_ESM.pdf]

## Table S2: Metrics from different assembly methods

| | | CLC Genomics Workbench | | | | | IDBA UD | | | | Metavelvet | | | |
|---|---|---|---|---|---|---|---|---|---|---|---|---|---|---|
| | | Contigs | Mean Length | Max Length | N50 | Percent In Contigs[b] | Contigs | Mean Length | Max Length | N50 | Contigs | Mean Length | Max Length | N50 |
| Donor 1 | Day 4 | **5457** | **947** | **41739** | **1444** | 94.5 | 7883 | 606 | 16701 | 1079 | 11990 | 332 | 5745 | 456 |
| | Day 8 | **3280** | **948** | **60551** | **1374** | 97.4 | 5624 | 506 | 20749 | 1036 | 29258 | 140 | 1859 | 229 |
| | Day 12 | **3926** | **1033** | **53146** | **1665** | 95 | 5199 | 686 | 28300 | 1340 | 10364 | 308 | 5535 | 1020 |
| | Day 16 | **4064** | **997** | **57967** | **1633** | 96.6 | 6433 | 578 | 18548 | 1196 | 10278 | 299 | 6743 | 664 |
| | Day 24 | **3383** | **939** | **63171** | 1691 | 96.9 | 5633 | 551 | 20249 | 1271 | 9596 | 279 | 6459 | **2414** |
| | Stool | **1879** | **829** | **29621** | **1069** | 96.1 | 2830 | 418 | 17331 | 809 | 14503 | 141 | 2773 | 191 |
| Donor 2 | Day 4 | **1946** | **686** | **38124** | **709** | 96.8 | 3246 | 345 | 18417 | 574 | 13622 | 148 | 1388 | 181 |
| | Day 8 | **4206** | **790** | **25757** | **963** | 96.6 | 6925 | 424 | 16493 | 746 | 6470 | 361 | 6457 | 562 |
| | Day 12 | **3201** | **773** | **33183** | **895** | 95.5 | 5912 | 380 | 12411 | 687 | 20112 | 160 | 1729 | 227 |
| | Day 16 | **5698** | **1025** | **53789** | **1667** | 97 | 9203 | 611 | 19208 | 1216 | 12318 | 386 | 6255 | 706 |
| | Day 24 | **6022** | **998** | **51019** | **1662** | 96.2 | 9030 | 599 | 15057 | 1132 | 15011 | 334 | 6067 | 98 |
| | Stool | **3739** | **1014** | **52910** | **1826** | 92.4 | 4188 | 661 | 27868 | 1503 | 10381 | 295 | 6174 | 1632 |
| Donor 8 | Day 3 | **6541** | **846** | **49980** | **1067** | 95.3 | 6723 | 544 | 15285 | 869 | 11236 | 335 | 4993 | 321 |
| | Day 6 | 4547 | **821** | **43340** | **975** | 95.3 | **3693** | 533 | 18075 | 836 | 29526 | 155 | 1916 | 183 |
| | Day 12 | **5781** | **890** | **49984** | **1222** | 95 | 6096 | 588 | 16130 | 982 | 10583 | 356 | 7480 | 506 |
| | Day 18 | **1114** | **876** | **48541** | **1550** | 98.2 | 3674 | 283 | 21185 | 568 | 14379 | 117 | 1615 | 171 |
| | Day 24 | **1018** | **815** | **48504** | **1264** | 98.8 | 3299 | 260 | 15020 | 423 | 10899 | 124 | 2275 | 326 |
| | Stool | **1266** | **1102** | **19694** | **2226** | 96.6 | 3576 | 415 | 14004 | 1138 | 17949 | 115 | 3478 | 114 |
| Donor 9 | Day 3 | **3391** | **827** | **69536** | **1074** | 97 | 4732 | 530 | 21652 | 971 | 24712 | 149 | 1659 | 68 |
| | Day 6 | **2425** | **829** | **45528** | **1083** | 98 | 4271 | 415 | 34999 | 781 | 20256 | 138 | 1562 | 199 |
| | Day 12 | **3556** | **901** | **94115** | **1179** | 97.1 | 3749 | 495 | 13218 | 779 | 26927 | 150 | 1884 | 244 |
| | Day 18 | **2867** | **909** | **93797** | **1272** | 97 | 3672 | 501 | 13588 | 886 | 23077 | 148 | 2454 | 112 |
| | Day 24 | **2913** | **649** | **93414** | **735** | 96.5 | 3332 | 361 | 9701 | 632 | 17927 | 148 | 2013 | 134 |
| | Stool | **2696** | **924** | **41934** | **1421** | 94.7 | 3090 | 530 | 32413 | 1084 | 5600 | 337 | 7040 | 100 |
| Donor 10 | Day 4 | **4533** | **1219** | **51727** | **2722** | 96 | 8707 | 607 | 16925 | 1348 | 12931 | 366 | 6753 | 146 |
| | Day 8 | **5148** | **927** | **40455** | **1241** | 97.1 | 6454 | 500 | 17015 | 825 | 9956 | 358 | 6084 | 428 |
| | Day 12 | **5891** | **1107** | **48332** | 1858 | 96.1 | 6643 | 625 | 29283 | 1058 | 14100 | 380 | 6188 | **2250** |
| | Day 16 | **5365** | **931** | **40497** | **1262** | 96.2 | 5674 | 548 | 14373 | 861 | 10566 | 351 | 7931 | 1140 |
| | Day 24 | **4808** | **1015** | **40172** | **1614** | 97.9 | 7070 | 564 | 17794 | 1019 | 10720 | 390 | 9062 | 987 |
| | Stool | 3364 | **956** | **50410** | **1578** | 97.6 | **3264** | 599 | 27849 | 1353 | 8530 | 281 | 9951 | 719 |

[a]The method with the least number of contigs, highest mean length, highest maximum length, and/or highest N50 values is represented in bold.

[b]Percentage of reads that were assembled into contigs
