# Supplementary material for: Chemostat culture systems support diverse bacteriophage communities from human feces
Source: Microbiome. 2015 Nov 9;3:58. doi: 10.1186/s40168-015-0124-3 (PMC4638026; doi:10.1186/s40168-015-0124-3)
Supplement: Additional file 3: — Supplemental figures. [file 40168_2015_124_MOESM3_ESM.pdf]

Figure S1

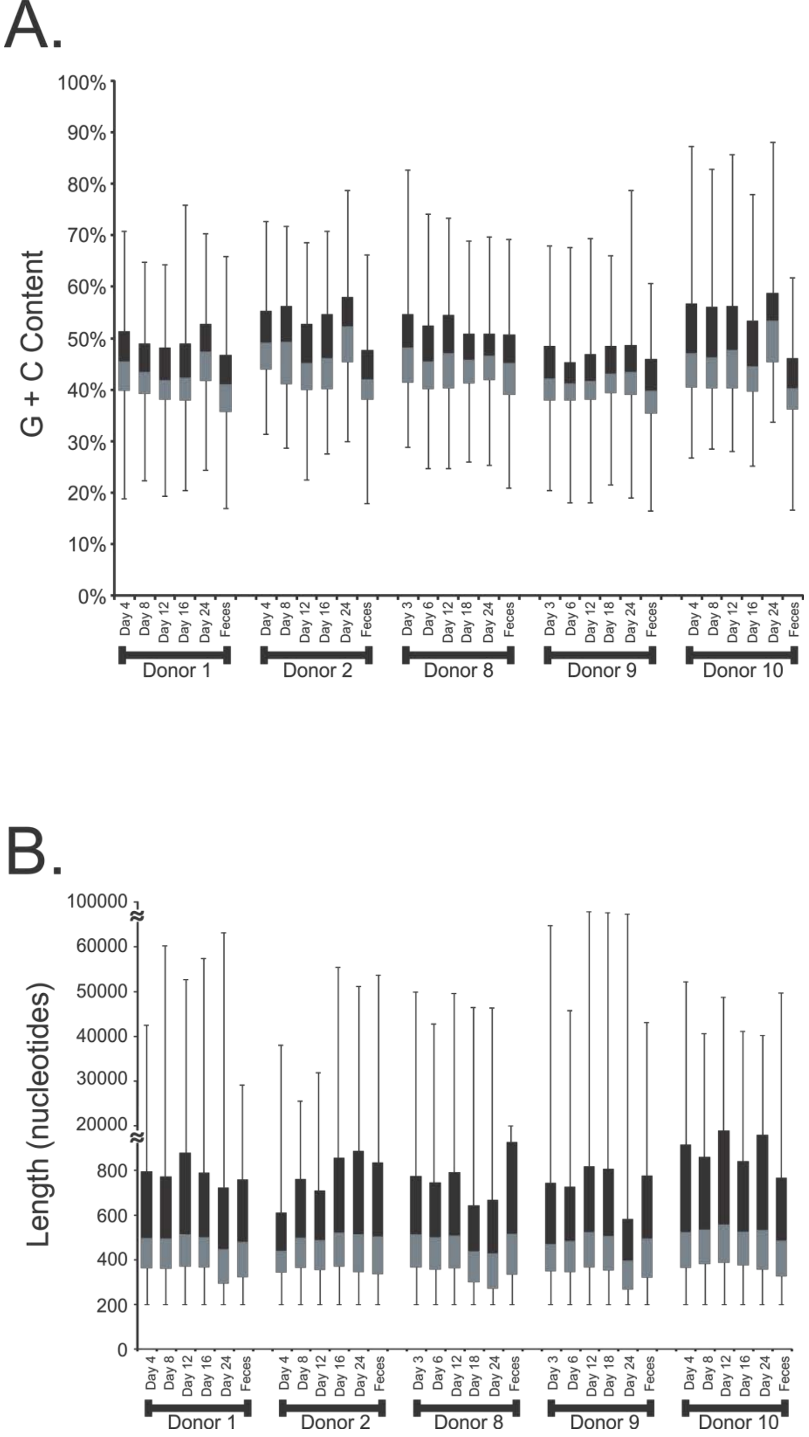

Figure S1: Box and Whiskers plots of median GC content (Panel A) and length (Panel B) of viral contigs for all chemostat virome time points and fecal viromes. Percent G + C content or length is shown on the y-axis and each donor and time point is shown on the x-axis.

Figure S2

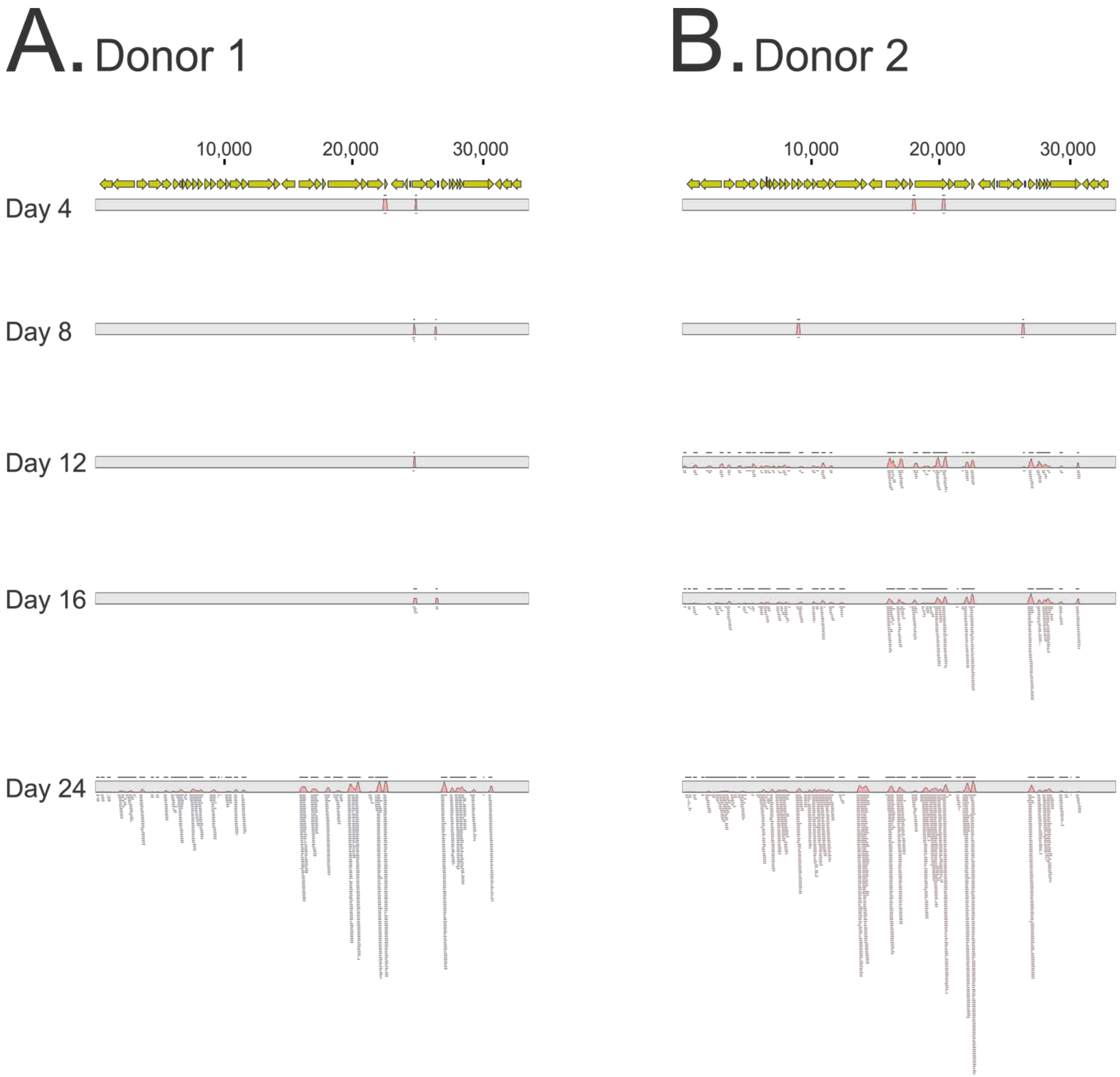

Figure S2: Read mappings of chemostat viromes to Enterobacteria Phage FIAA91ss. Panel A represents donor #1 and Panel B represents donor #2. The genes and their respective directions are shown by the yellow arrows represented above. Read mappings from each day are shown for both donors #1 and #2. The relative locations along the phage genomes are demonstrated by the scale at the top of the diagram, and the relative proportion of reads mapping to the phage are shown at the lower portion of the diagram.

Figure S3

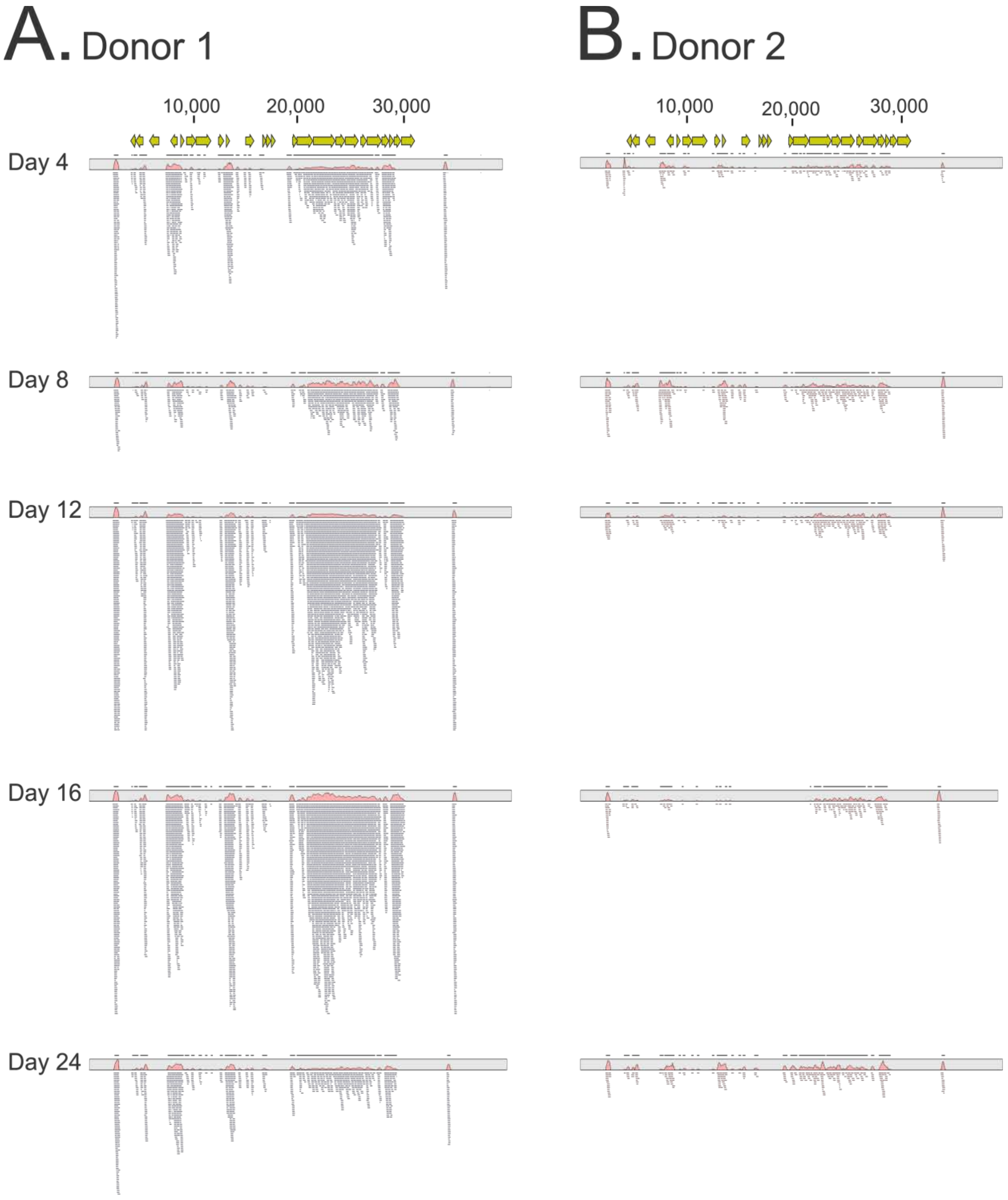

Figure S3: Read mappings of chemostat viromes to Enterobacteria Phage IME10. Panel A represents donor #1 and Panel B represents donor #2. The genes and their respective directions are shown by the yellow arrows represented above. Read mappings from each day are shown for both donors #1 and #2. The relative location along the phage genomes are demonstrated by the scale at the top of the diagram, and the relative proportion of reads mapping to the phage are shown at the lower portion of the diagram.

Figure S4

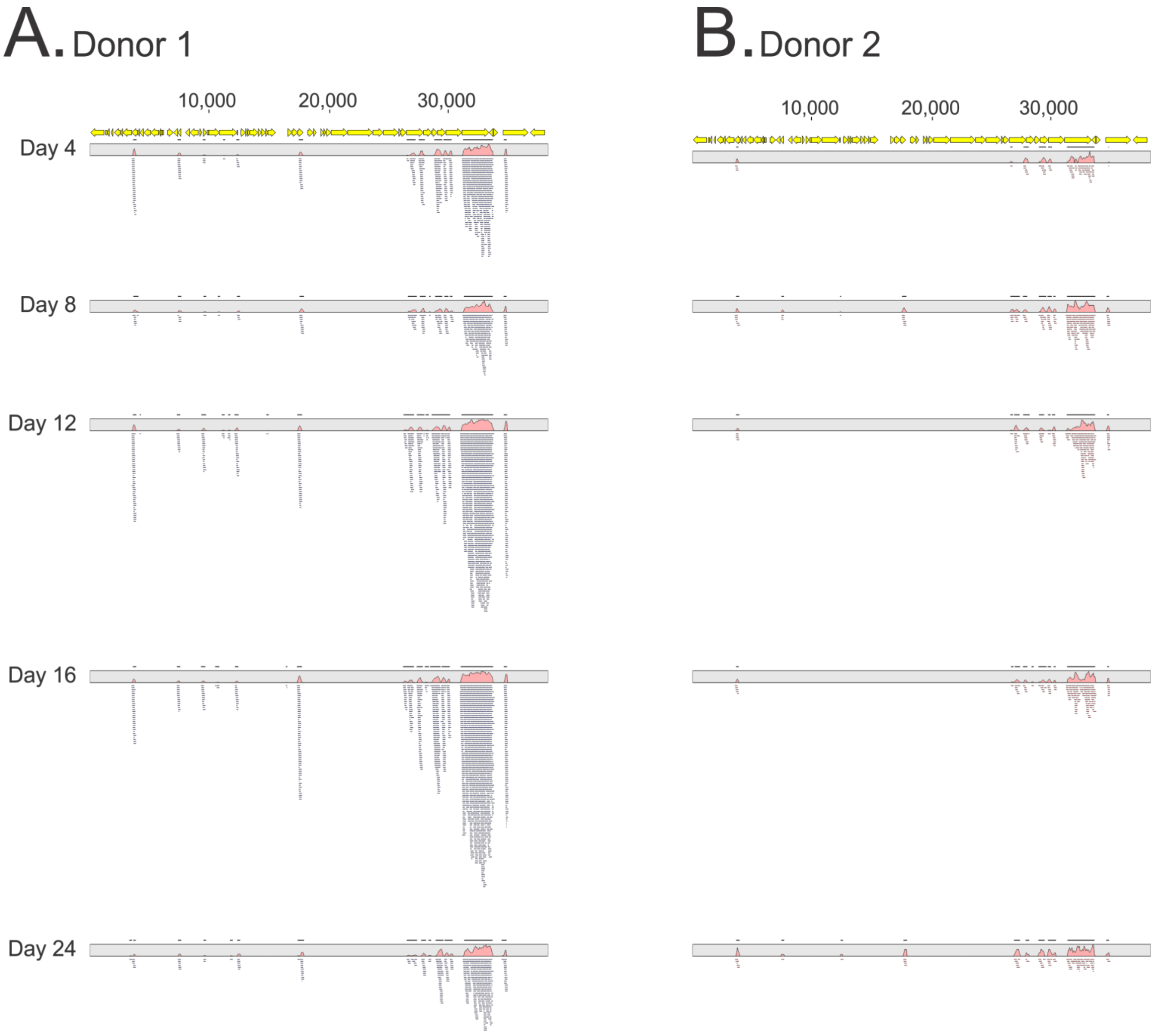

Figure S4: Read mappings of chemostat viromes to Enterobacteria Phage HK620. Panel A represents donor #1 and Panel B represents donor #2. The genes and their respective directions are shown by the yellow arrows represented above. Read mappings from each day are shown for both donors #1 and #2. The relative location along the phage genomes are demonstrated by the scale at the top of the diagram, and the relative proportion of reads mapping to the phage are shown at the lower portion of the diagram.

Figure S5

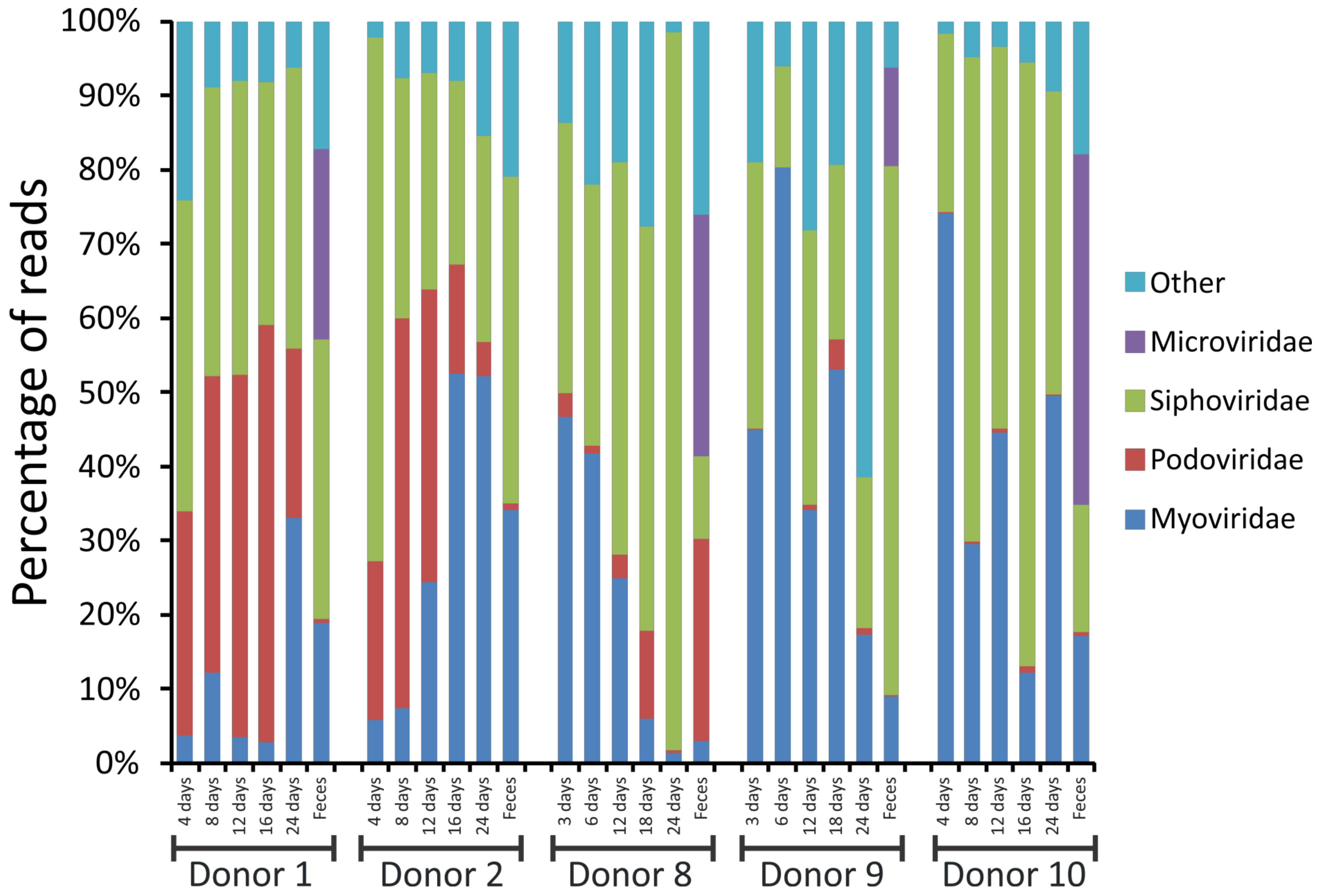

Figure S5: Bar graphs demonstrating the proportion of reads significantly similar to known bacteriophage families in feces and chemostat material. Each bar represents the day of culture, and the last bar for each donor represents fecal viromes.

Figure S6

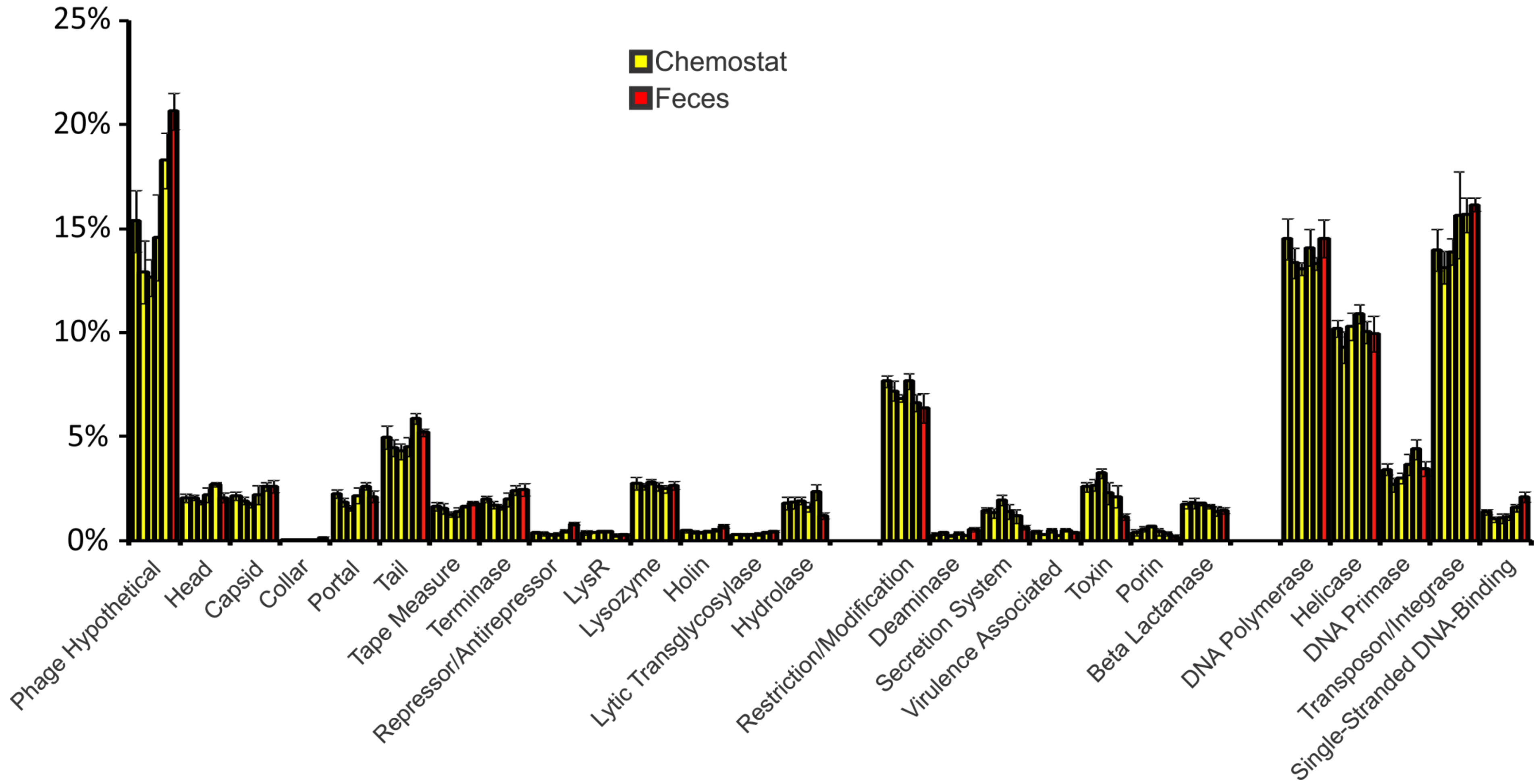

Figure S6: Bar graph of the percentage of contigs (± standard deviation) with phage homologues in the NR database from the fecal and chemostat viromes of all subjects. The annotation of each homologue is shown on the x-axis and the y-axis represents the percentage of contigs. Each yellow bar from left to right represent chemostat viromes from days 4, 8, 12, 16, and 24 of culture for donors #1, #2, and #10. The chemostat viromes from donors #8 and #9 were sampled on days 3, 6, 12, 18, and 24. The red bar represents the fecal viromes.

Figure S7

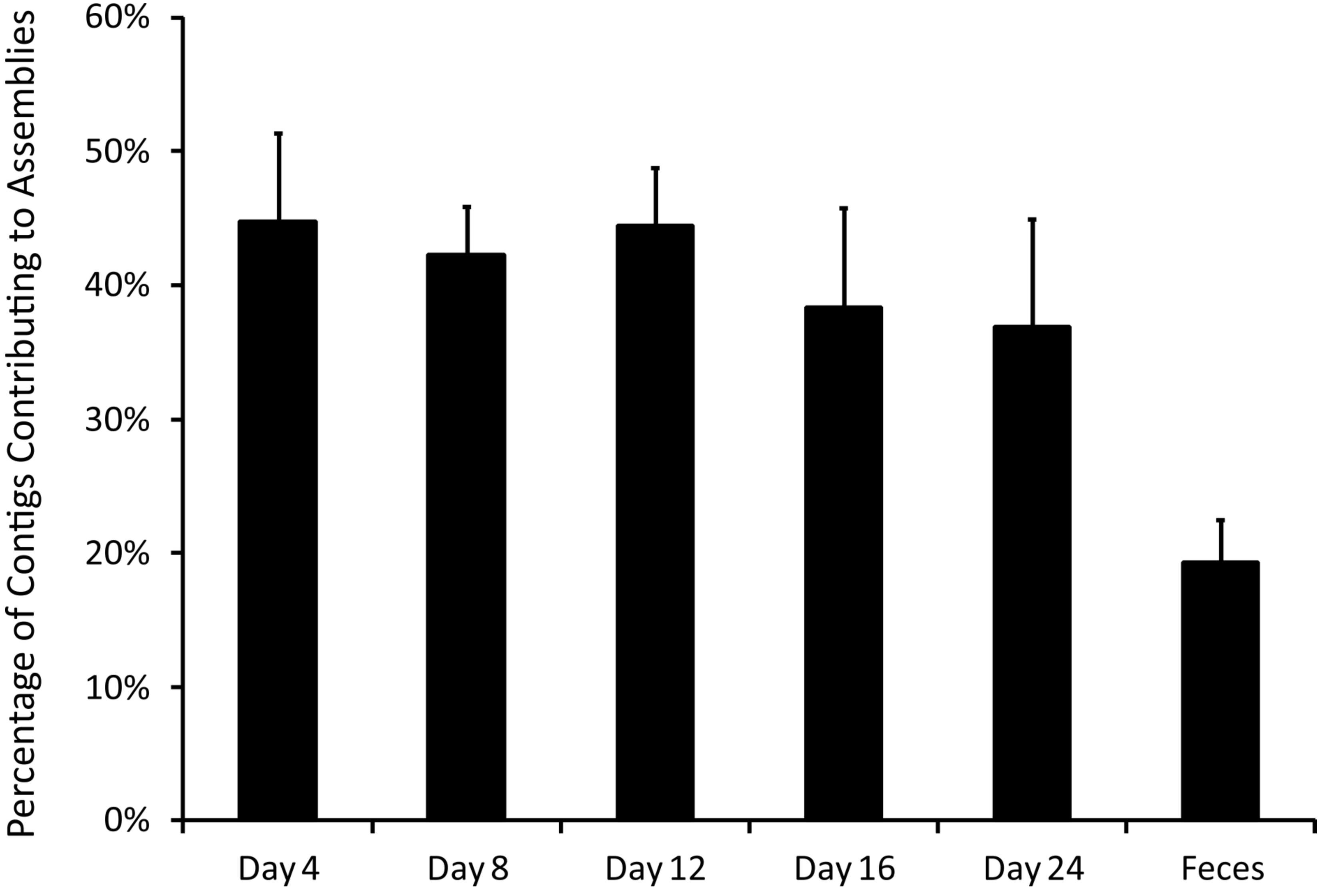

Figure S7: Bar graph of the percentage (± standard deviation) of contigs used in the construction of assemblies from all time points in each donor (Panel A). The contigs from all donors were used to construct assemblies and the proportion of all assemblies that contained contigs from each time point from each donors is demonstrated on the y-axis.

Figure S8

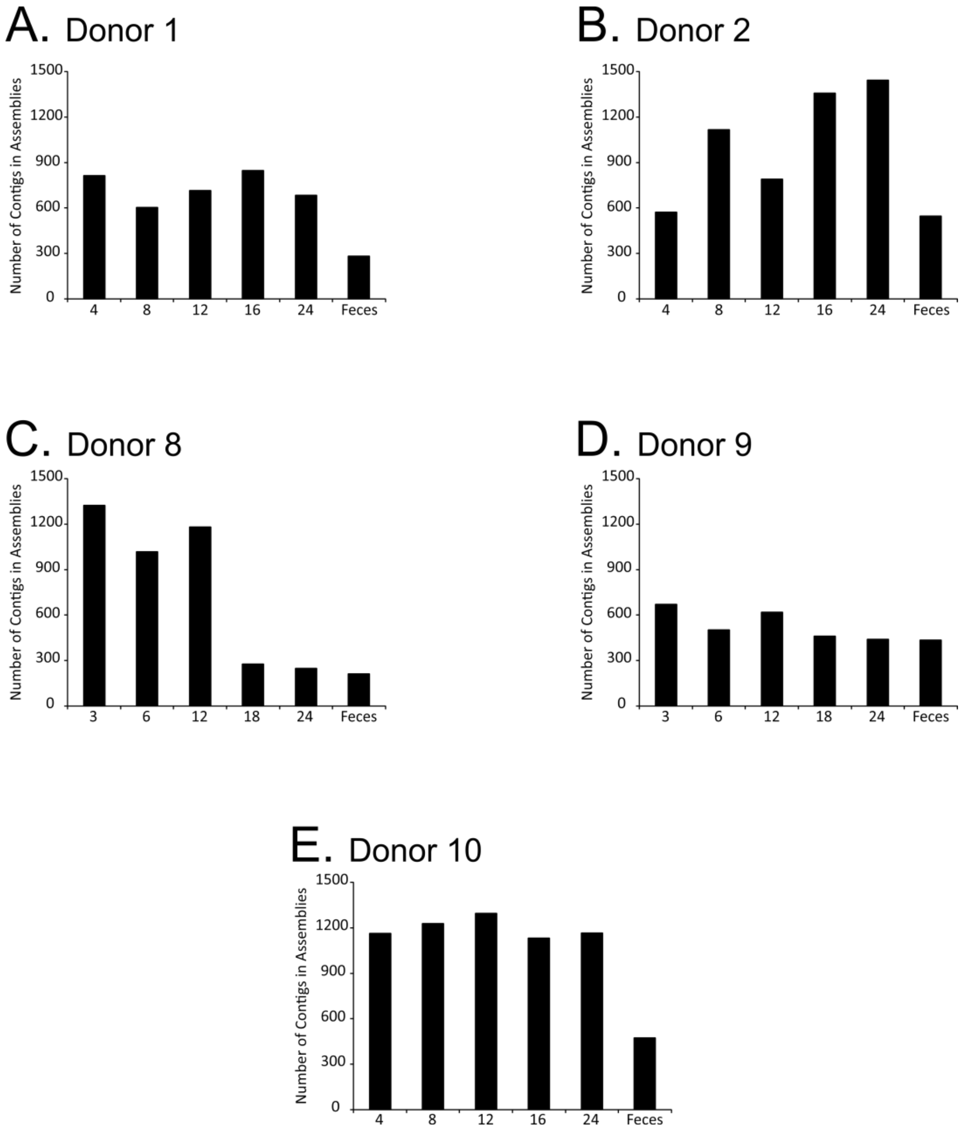

Figure S8: Bar graphs of the number of contigs used in the construction of assemblies from all time points in each donor. The number of contigs is represented on the y-axis, and the time point (in days) or the sample type is shown on the x-axis.

Figure S9

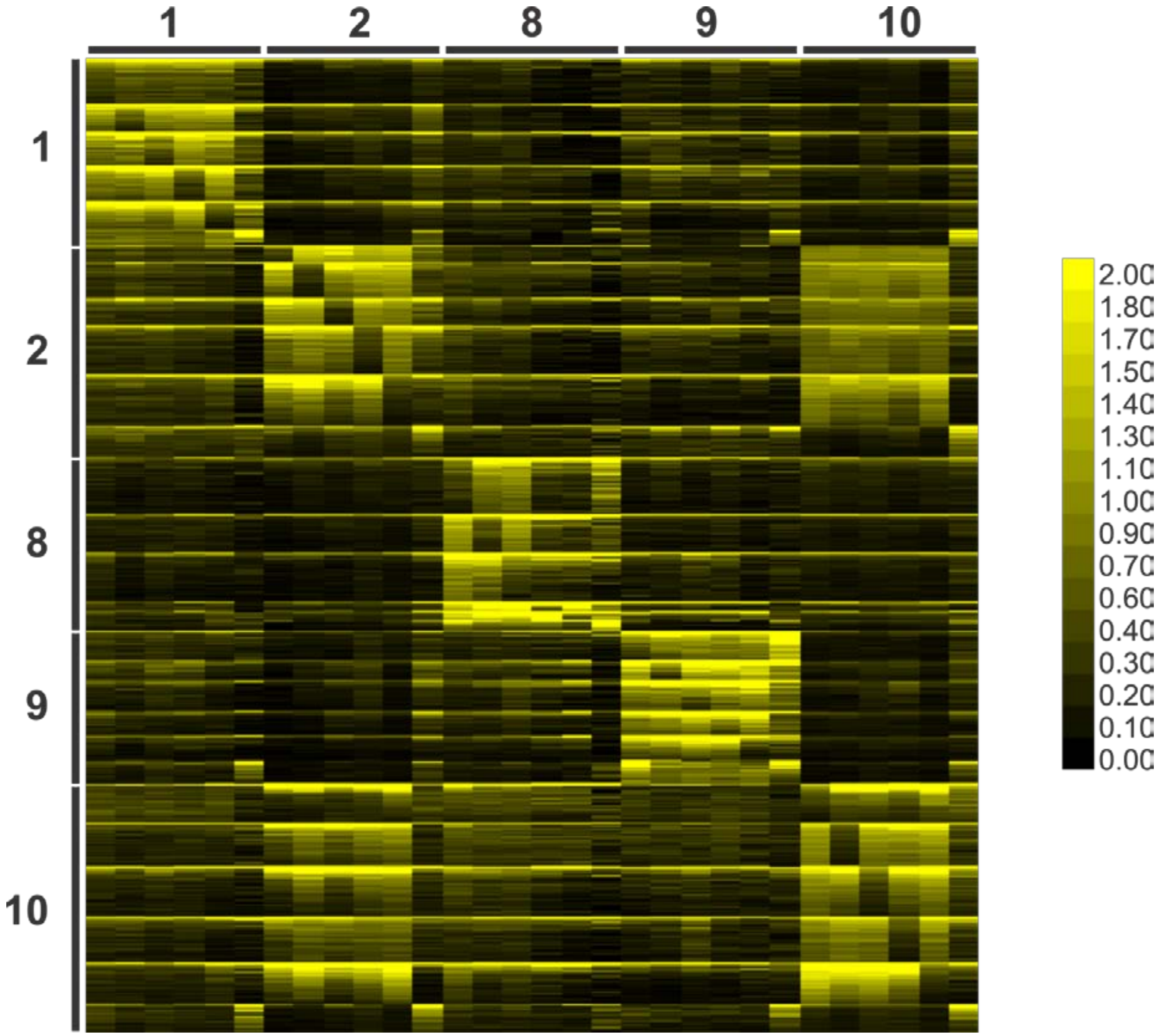

Figure S9: Heatmap of shared contigs across all donors and time points. The heatmap is organized by donor and time point, where the donor# is denoted along each axis, and the individual time points are denoted by each column consecutively from day 4 to day 24 left to right for each donor. The last column for each donor represents the feces. Each row represents an individual contig, and rows are ordered consecutively across each subject from contigs identified on day 4 to the feces. The ‘matrix-like’ appearance of the heatmap is due to the high intensity of similar contigs across all time points within each donor.

Figure S10

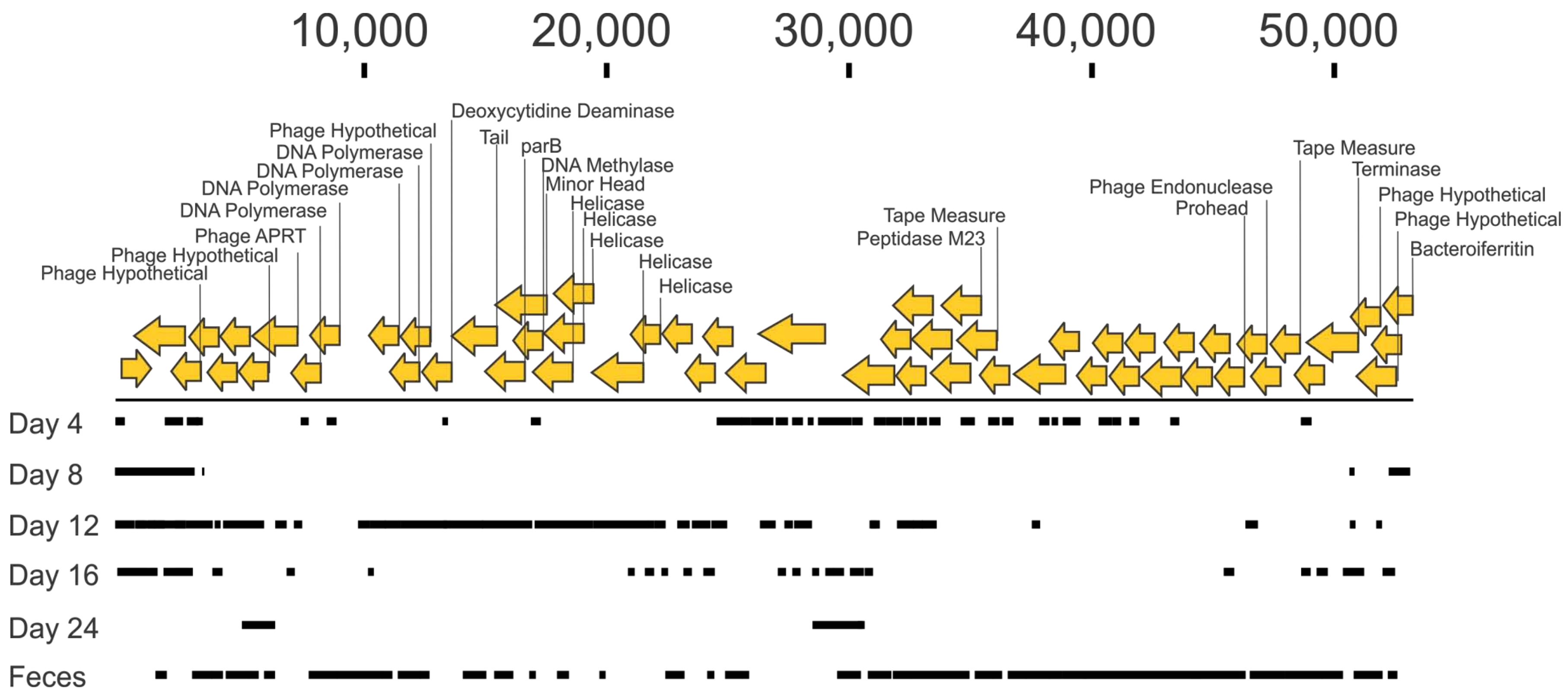

Figure S10: Assembly of contig 78 from all time points in donor #1. The portions of the contig identified in each time point or the feces are represented by the colored boxes. Putative ORFs and their directions are represented by the yellow arrows and their annotations are represented above. The length of the contig is denoted at the top.

Figure S11

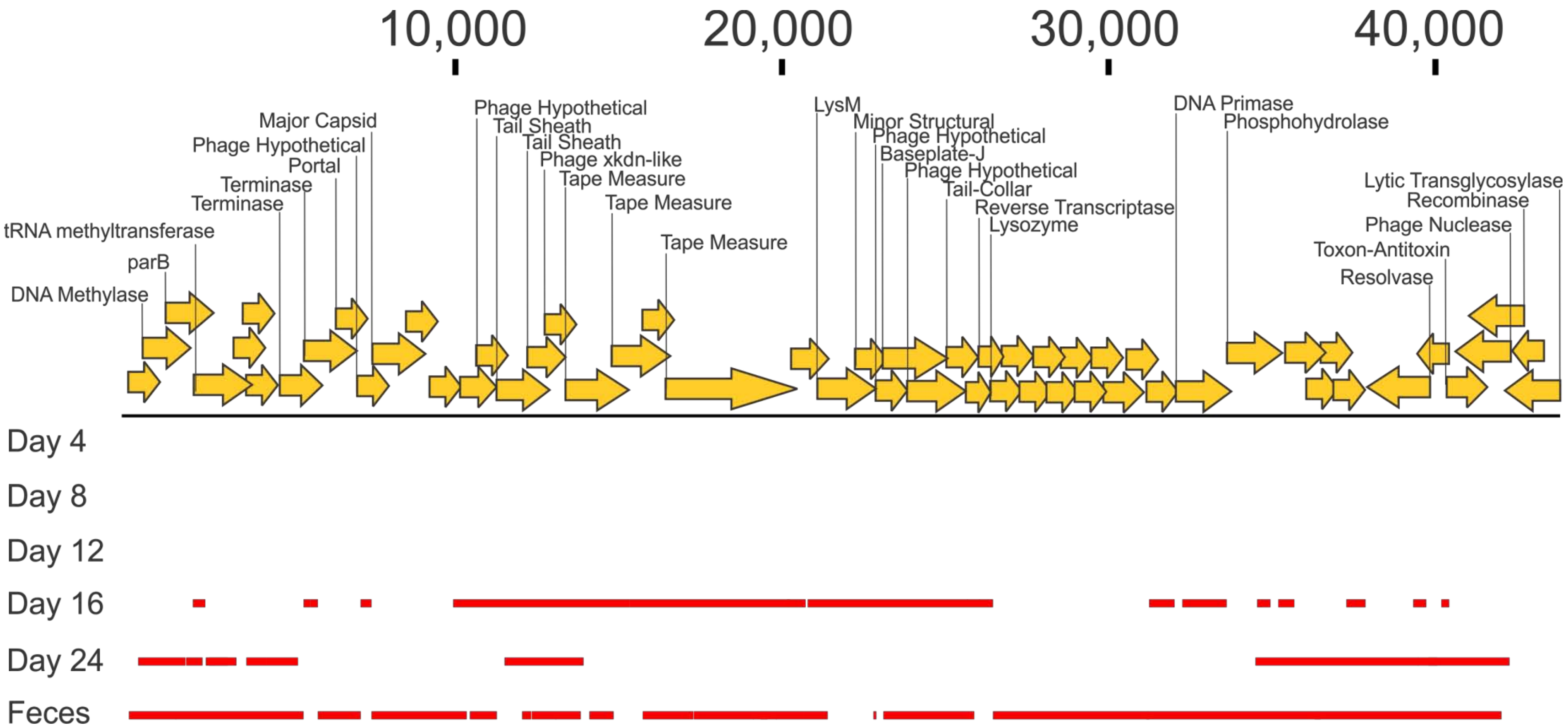

Figure S11: Assembly of contig 38 from all time points in donor #2. The portions of the contig identified in each time point or the feces are represented by the colored boxes. Putative ORFs and their directions are represented by the yellow arrows and their annotations are represented above. The length of the contig is denoted at the top.

Figure S12

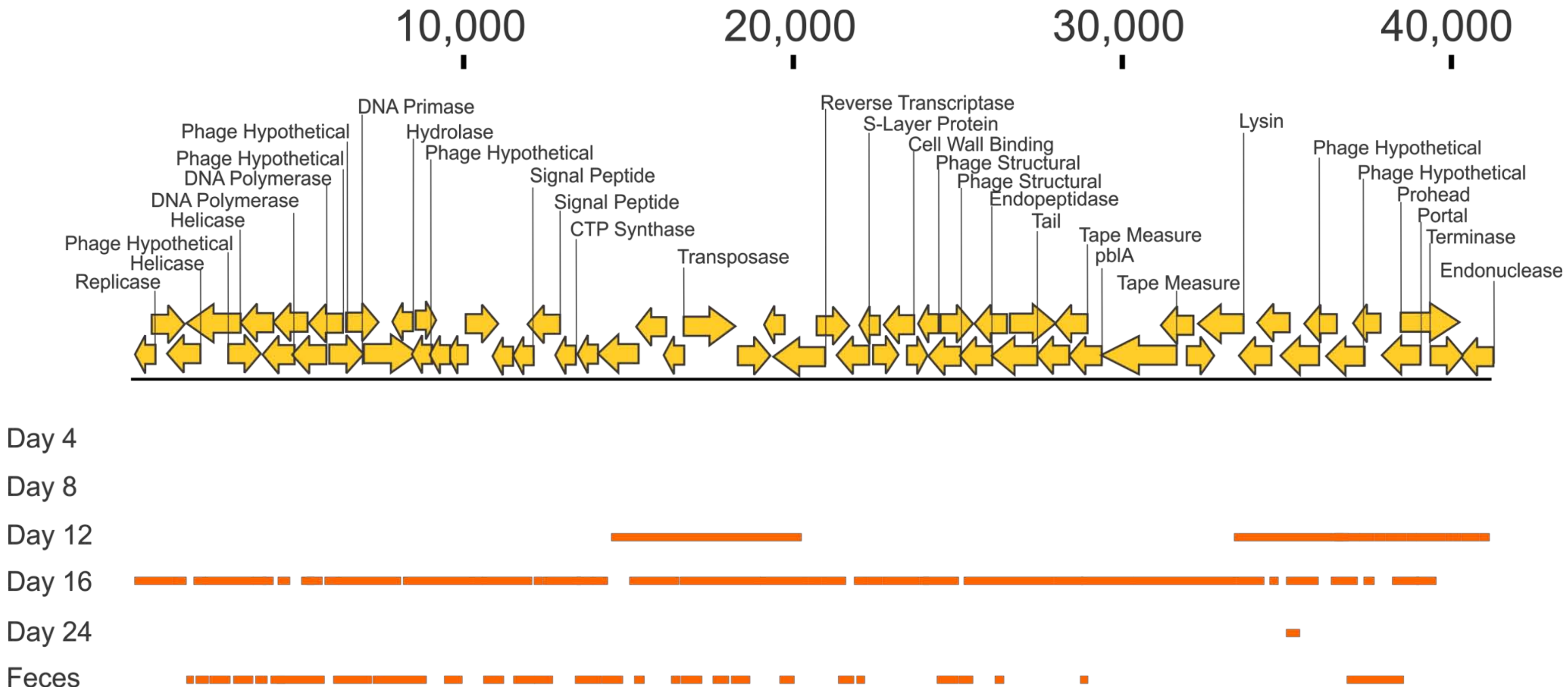

Figure S12: Assembly of contig 61 from all time points in donor #10. The portions of the contig identified in each time point or the feces are represented by the colored boxes. Putative ORFs and their directions are represented by the yellow arrows and their annotations are represented above. The length of the contig is denoted at the top.

Figure S13

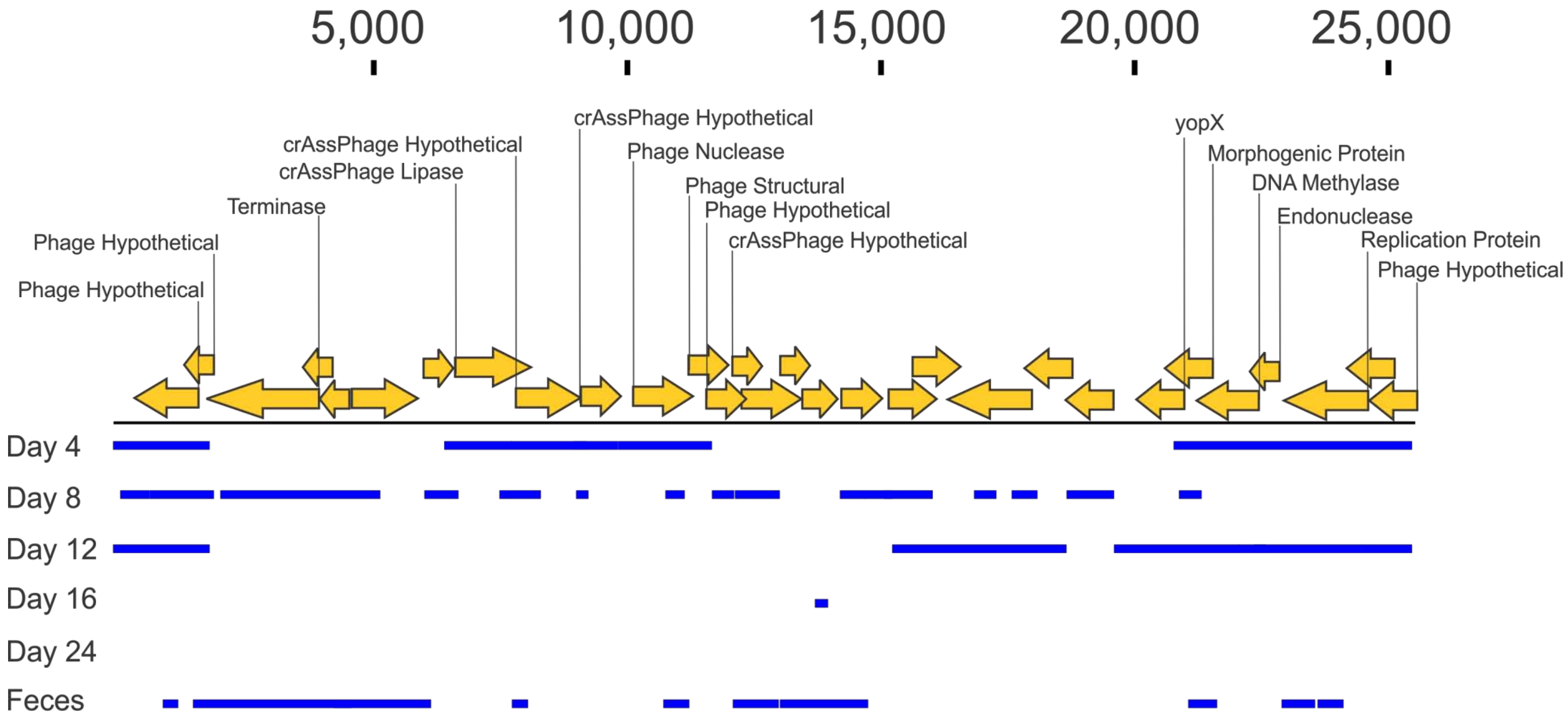

Figure S13: Assembly of contig 318 from all time points in donor #8. The portions of the contig identified in each time point or the feces are represented by the colored boxes. Putative ORFs and their directions are represented by the yellow arrows and their annotations are represented above. The length of the contig is denoted at the top.

Figure S14

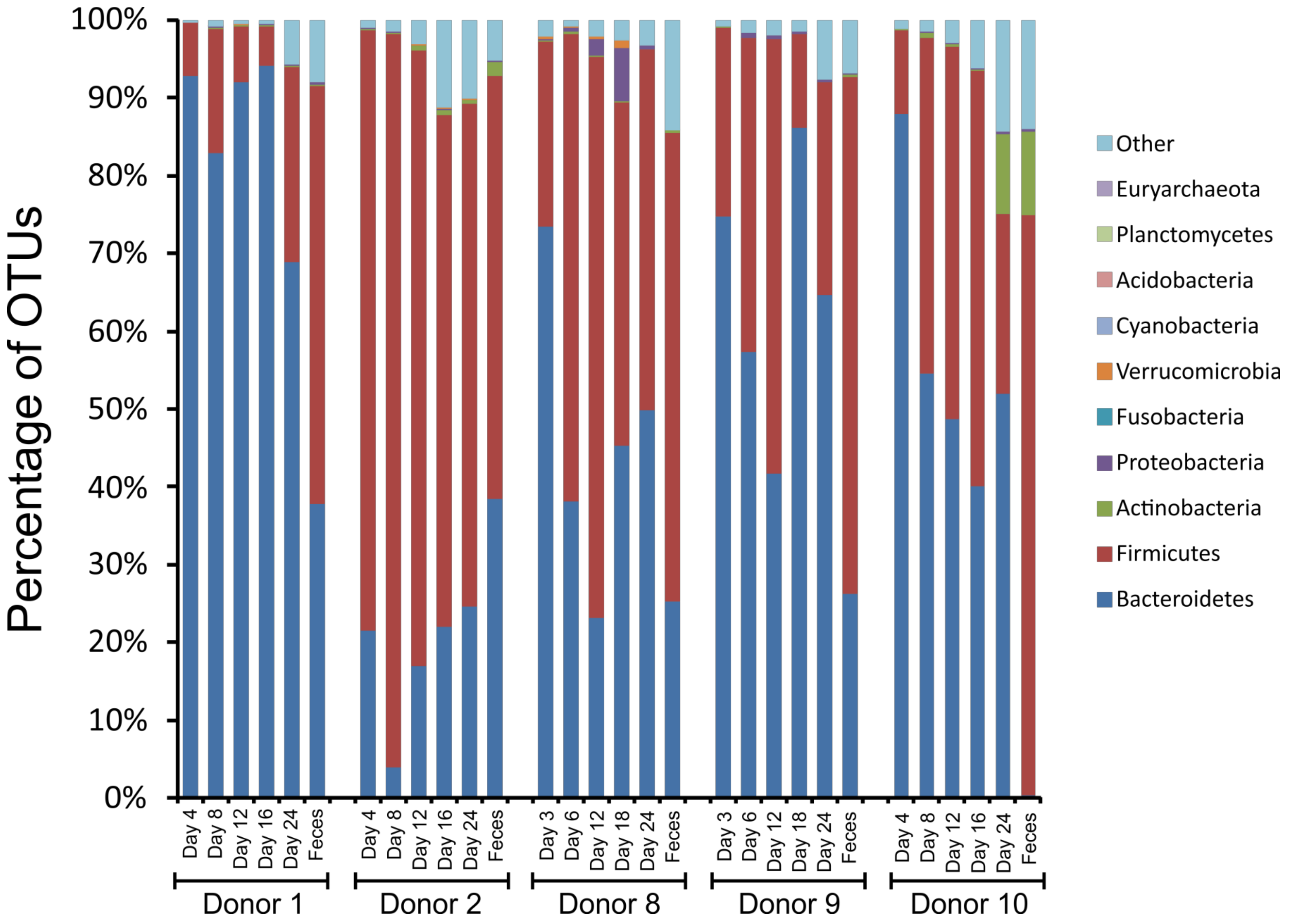

Figure S14: Bar graphs representing bacterial taxonomy based on 16S rRNA genes at the Phylum level for feces and chemostat cultures. Each bar represents the day of culture, and the last bar for each donor represents fecal viromes.

Figure S15

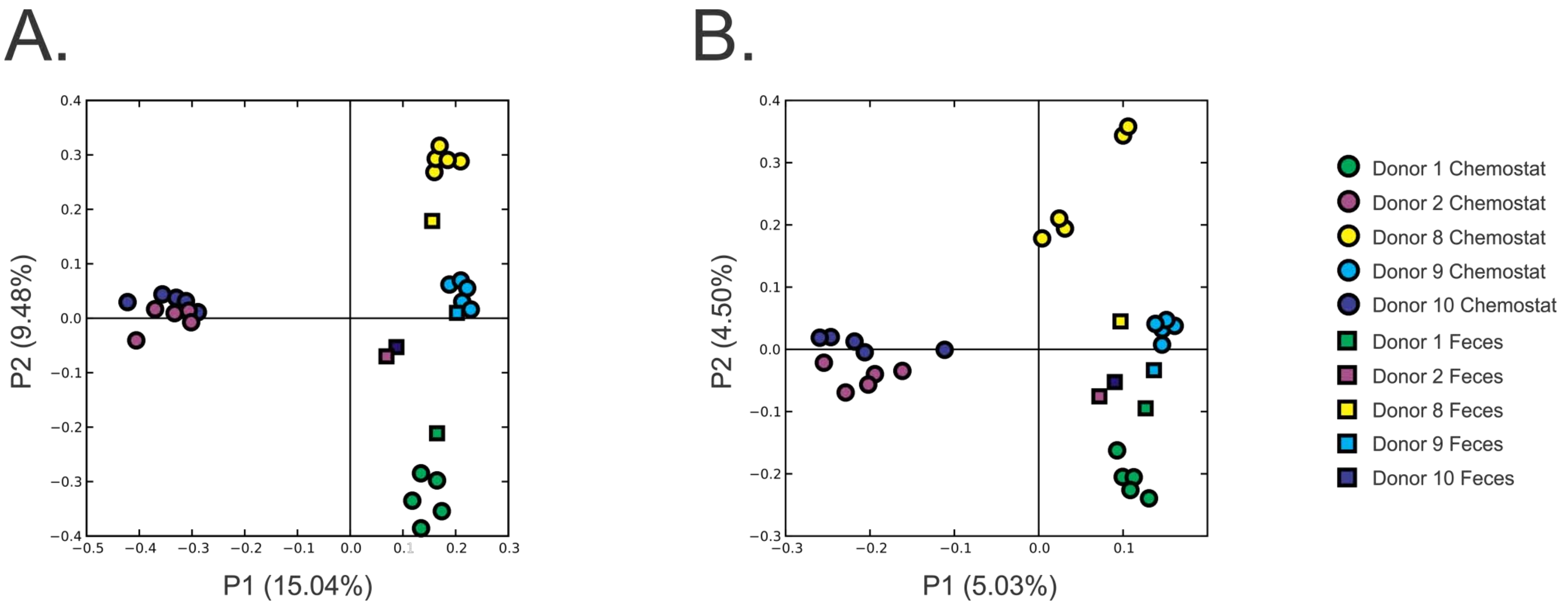

Figure S15: Principal coordinates analysis of beta diversity present in viromes based on the contributions from each donor and time point to assemblies (Panel A) and BLASTX best-hit profiles (Panel B). Fecal samples are represented by squares and chemostat viromes are represented by circles.

Figure S16

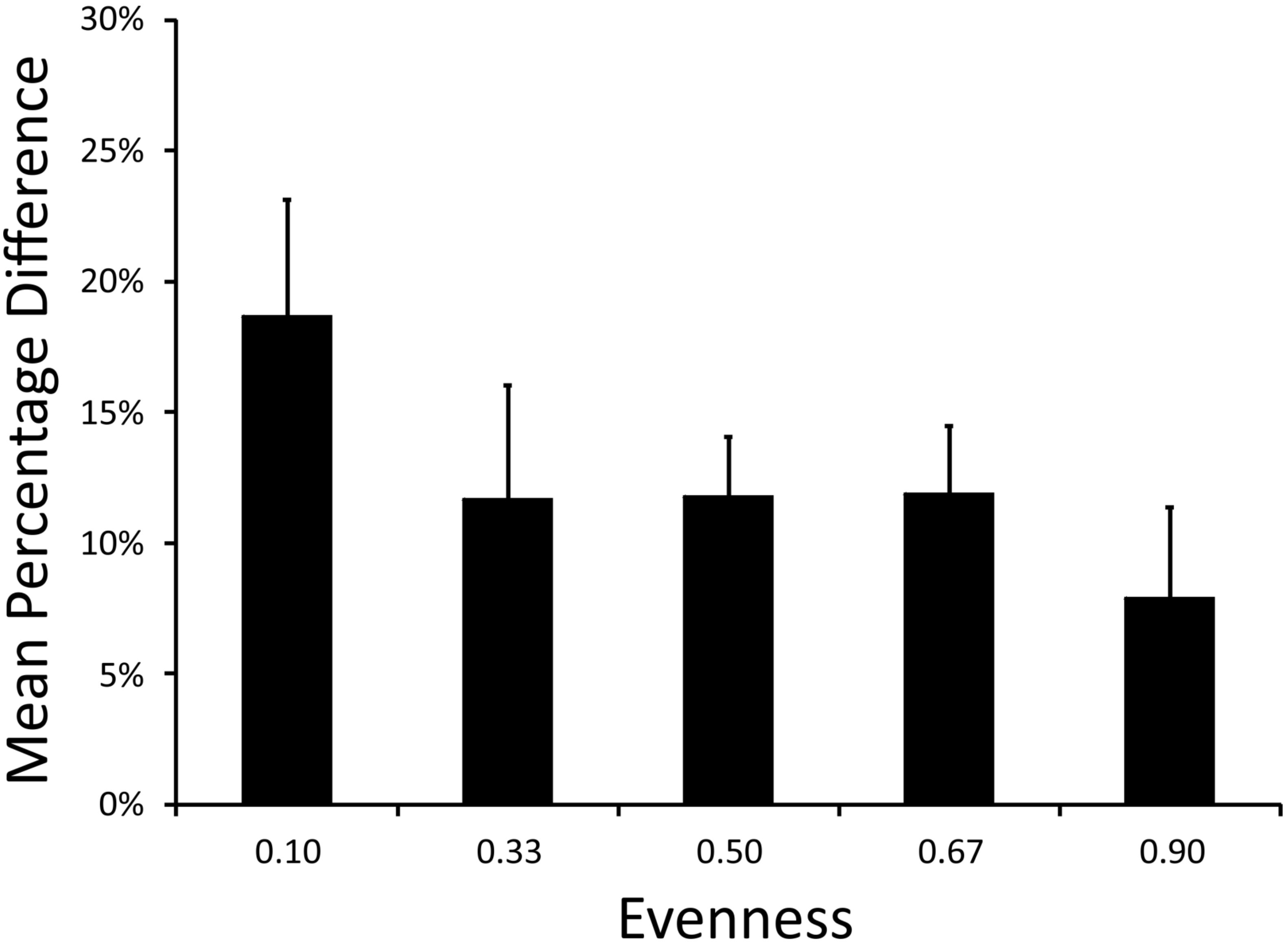

Figure S16: Bar graphs (±standard deviation) demonstrating the mean differences between measurements of the Shannon Index and the HVDI by evenness level. The mean percentage difference is shown on the y-axis and the evenness values are shown on the x-axis.

Figure S17

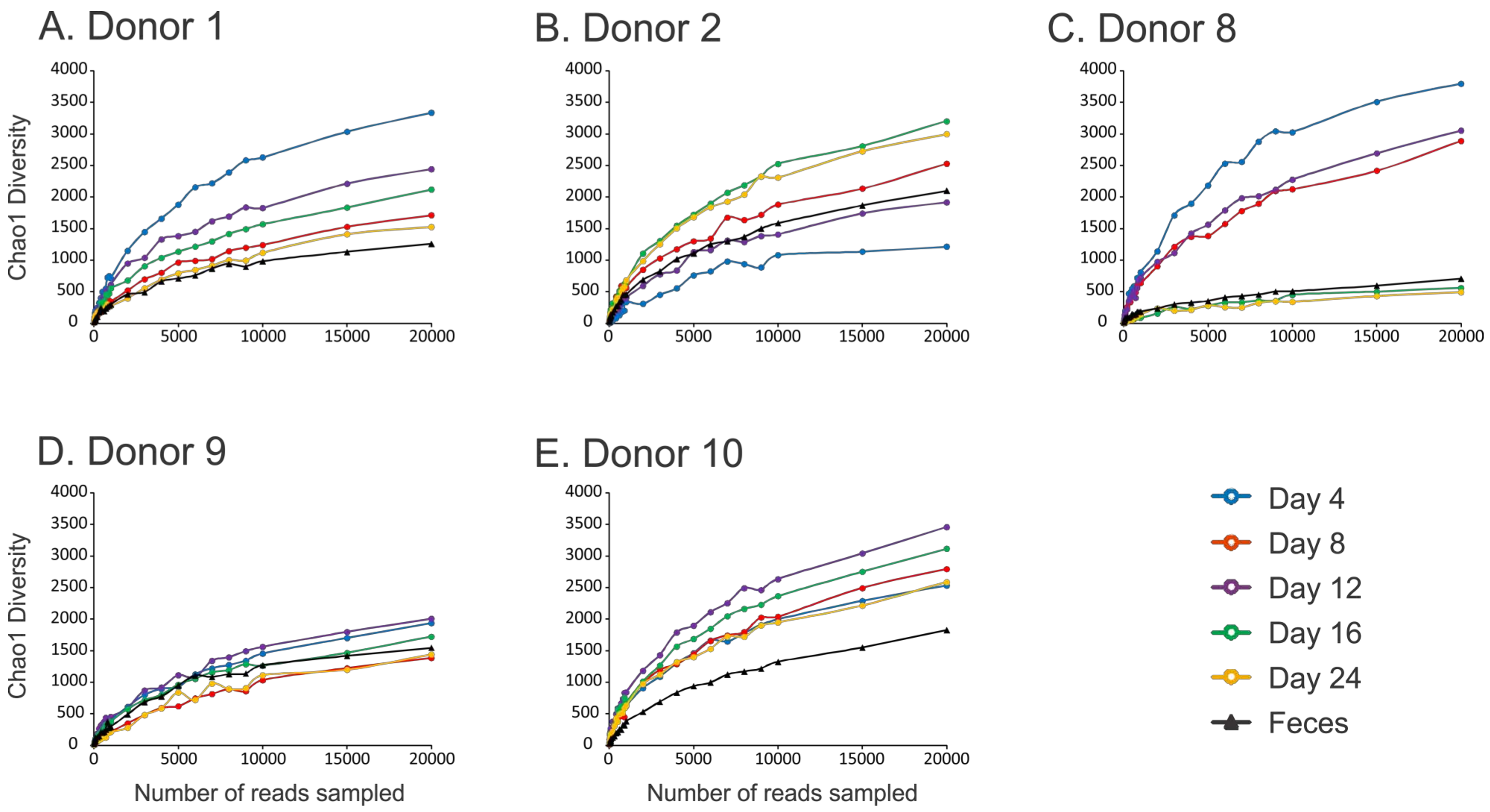

Figure S17: Rarefaction analysis of alpha diversity based on homologous diversity for fecal and chemostat viromes from all donors. The number of reads sampled is represented on the x-axis, and the homologous diversity index (based on the chao1 index) is represented on the y-axis. For donors #1, #2, and #10, chemostat viromes from day 4 are shown in cyan, day 8 in red, day 12 in purple, day 16 in green, and day 24 in yellow. For donors #8, and #9, chemostat viromes from day 3 are shown in cyan, day 6 in red, day 12 in purple, day 18 in green, and day 24 in yellow. Fecal samples are represented in black.

Figure S18

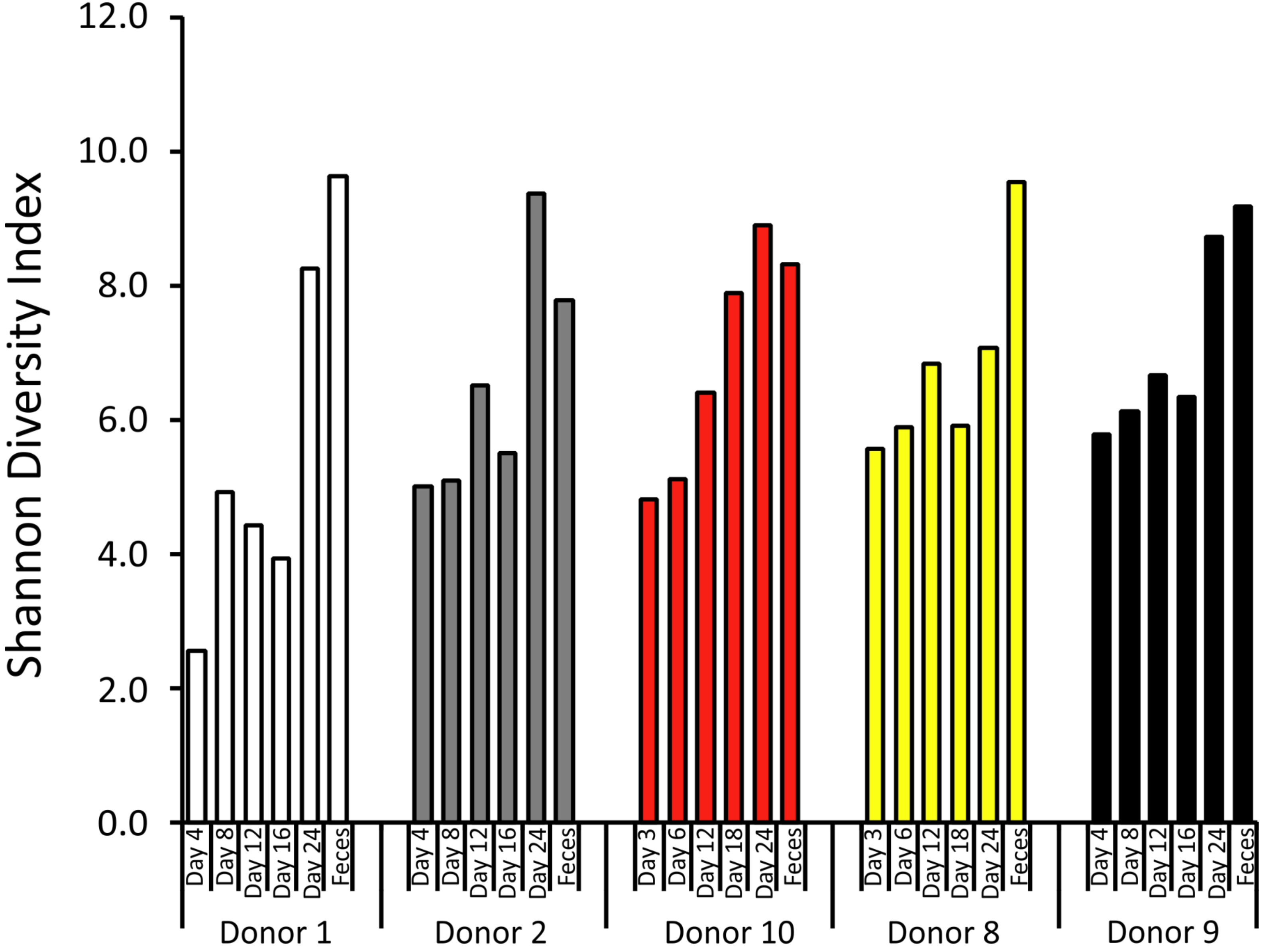

Figure S18: Bar graphs representing the Shannon Diversity Index for the bacterial taxonomy based on 16S rRNA genes for all subjects. The y-axis represents diversity, and on the x-axis each bar from left to right represents day of culture. The last bar for each donor represents the fecal viromes.
